# Decoding MUC1 and AR axis in a radiation-induced neuroendocrine prostate cancer cell-subpopulation unveils novel therapeutic targets

**DOI:** 10.1038/s41420-025-02597-4

**Published:** 2025-07-03

**Authors:** Catarina Macedo-Silva, Ângela Albuquerque-Castro, Iris Carriço, Joana Lencart, Isa Carneiro, Lucia Altucci, João Lobo, Vera Miranda-Gonçalves, Rui Henrique, Margareta P. Correia, Carmen Jerónimo

**Affiliations:** 1 Cancer Biology & Epigenetics Group, IPO Porto Research Center (CI-IPOP) / CI-IPOP@ RISE, Portuguese Oncology Institute of Porto (IPO Porto) / Porto Comprehensive Cancer Center Raquel Seruca (Porto.CCC), R. Dr. António Bernardino de Almeida, Porto, Portugal; 2 Doctoral Program in Pathology and Molecular Genetics, ICBAS-School of Medicine and Biomedical Sciences, University of Porto (ICBAS-UP), Rua Jorge Viterbo Ferreira 228, Porto, Portugal; 3 Medical Physics, Radiobiology and Radiation Protection Group - IPO Porto Research Center (CI-IPOP) / CI-IPOP@RISE, Portuguese Oncology Institute of Porto (IPO Porto) / Porto Comprehensive Cancer Center Raquel Seruca (Porto.CCC), R. Dr. António Bernardino de Almeida, Porto, Portugal; 4 Department of Medical Physics, Portuguese Oncology Institute of Porto (IPO Porto) / Porto Comprehensive Cancer Center Raquel Seruca (Porto.CCC), R. Dr. António Bernardino de Almeida, Porto, Portugal; 5 Department of Pathology, Portuguese Oncology Institute of Porto (IPO Porto) / Porto Comprehensive Cancer Center Raquel Seruca (Porto.CCC), R. Dr. António Bernardino de Almeida, Porto, Portugal; 6 Department of Precision Medicine, University of Campania “Luigi Vanvitelli”, Naples, Italy; 7 BIOGEM, Molecular Biology and Genetics Research Institute, Avellino, Italy; 8 IEOS, Institute of Endocrinology and Oncology, Naples, Italy; 9 Department of Pathology and Molecular Immunology, ICBAS-School of Medicine and Biomedical Sciences, University of Porto, Rua Jorge de Viterbo Ferreira 228, Porto, Portugal

**Keywords:** Cancer therapeutic resistance, Prostate cancer

## Abstract

Despite the initial efficacy of radiotherapy (RT) in treating prostate adenocarcinoma (PCa), disease progression can lead to the emergence of neuroendocrine prostate cancer (NEPC) - a highly aggressive malignancy for which standard therapies are mostly ineffective. Although oncogenic *MUC1-C* is a leading driver of NEPC and of PCa lineage plasticity, its putative role in response to RT, including RT-induced neuroendocrine transdifferentiation (tNED), has not been explored. We thus aimed to explore the interplay between androgen receptor (AR) signaling and MUC1 in PCa progression to NEPC. Firstly, using a radioresistant PCa cell line (22Rv1-RR), we demonstrated that epigenetic suppression of AR signaling led to MUC1/MUC1-C upregulation, which seems to be activated through γSTAT3. MUC1 activation is positively associated with increased expression of neuroendocrine-related markers, including CD56, chromogranin A, synaptophysin, and INSM transcriptional repressor 1 (INSM1). In NEPC tissues and compared to prostate adenocarcinoma, MUC1 was upregulated and negatively correlated with AR, which was suppressed. Finally, proteomic analyses revealed that MUC1 activation upon RT selective pressure led to the acquisition of stemness features, induction of epithelial to mesenchymal transition, and enhancement of basal cell-like traits. Notably, MUC1 knockdown significantly boosted response to RT in both 22Rv1-RR and DU145 cell lines. Moreover, AR-induced overexpression in PC3 cell lines entailed MUC1 downregulation, resulting in attenuated neuroendocrine traits and radioresistance, as well as impaired cell migration and invasion capabilities. Collectively, these results highlight MUC1 as a promising radiosensitization target and may ultimately help overcome therapy resistance and NEPC progression.

## Introduction

Considering that nearly 90% of prostate cancers (PCa) are diagnosed as organ-confined disease, the long-standing challenge remains to overcome therapeutic resistance and disease progression to more, uncurable advanced stages. Although neuroendocrine prostate cancers (NEPC) represent a rare histological type, accounting for only 1% of all diagnosed PCa, focal treatment-induced neuroendocrine differentiation (NED) occurs in approximately 30% of advanced PCa [1–3]. Likewise, de novo neuroendocrine transdifferentiation (tNED) represents a common progression route for PCa, consistently associated with resistance to androgen deprivation (ADT) and/or radiotherapy (RT) [4, 5]. NEPC encompasses traits such as (i) loss of androgen receptor (AR) axis accompanied by low or absent levels of prostate-specific antigen (PSA), (ii) heightened stemness and (iii) increased cell aggressiveness associated with epithelial-mesenchymal transition (EMT) induction [6].

Tumors are complex ecosystems to which heterogeneity adds another level of challenge for the dissection of the underlying mechanisms. As a transcription factor, AR plays pivotal roles in PCa growth and progression. Currently, neoadjuvant or concurrent ADT combined with RT is an established standard-of-care for intermediate/high risk organ-confined PCa patients [7]. Despite the initial success of ADT in tumor reduction, PCa may rapidly relapse and adapt to progress to more advanced stages [8, 9]. The role of AR in PCa radioresistance is controversial and little has been explored in this field. AR has been shown to exhibit contrasting biological functions, initially acting as oncogenic driver and later as tumor suppressor [10]. Upon disease progression, AR reduction could be associated with a variety of adaptive resistance mechanisms, including enriched gene mutations and/or epigenetic modulation. Indeed, we have previously shown evidence of the involvement of epigenetic mechanisms in *AR* gene promoter regulation [11]. We found that although the AR negative cell line DU145 disclosed heterogeneity in *AR* methylation signature, AR re-expression was not successfully accomplished upon 5-aza-2-deoxycytidine (DAC) and Trichostatin A (TSA) exposure, due to the increase in repressive histone markers acting as alternative protective mechanism fostering cancer cell survival [11].

In clinical practice, AR heterogeneity and variations represents a serious concern that hampers PCa eradication. Hence, finding alternative oncogenic signaling pathways activated in the absence of AR is key to disclose new therapeutic avenues for advanced PCa, particularly in the context of RT resistance and treatment-induced NED. Remarkably, AR signaling suppression has been associated with oncogenic MUC1 overexpression, and with its cell surface functional fraction -MUC1-C [12]. Indeed, MUC1-C has been shown to drive NEPC progression, promoting self-renewal capacity and tumorigenicity [12]. Despite being mostly considered an oncogene, overexpressed in multiple solid tumors, MUC1 is also involved in metabolic reprograming of cancer cells, contributing to pancreatic cancer radioresistance [13]. Furthermore, MUC1 overexpression was also shown to confer radioresistance in head and neck cancer cells and hepatocellular carcinomas [14, 15]. Additionally, MUC1 was involved in important functions enabling cancer spread and overall cell aggressiveness [16]. Nonetheless, a putative role in the acquisition of PCa radioresistance has not been studied, yet.

Hence, we sought to explore the AR-MUC1 signaling axis in a PCa radiation-resistant cell line, which we previously established [17], to dissect PCa radioresistant mechanisms and uncover novel therapeutic targets. We found that, in RR PCa cells, overexpression of MUC1-C due to AR abrogation led to increased invasiveness and aggressiveness of neoplastic cells. Overall, MUC1 emerged as a promising target for radiosensitization, particularly in patients with advanced androgen-insensitive and unresponsive PCa.

## Results

### Mucin 1 (MUC1) is increased in neuroendocrine-like PCa cells exhibiting radioresistance

**Fig. 1:**
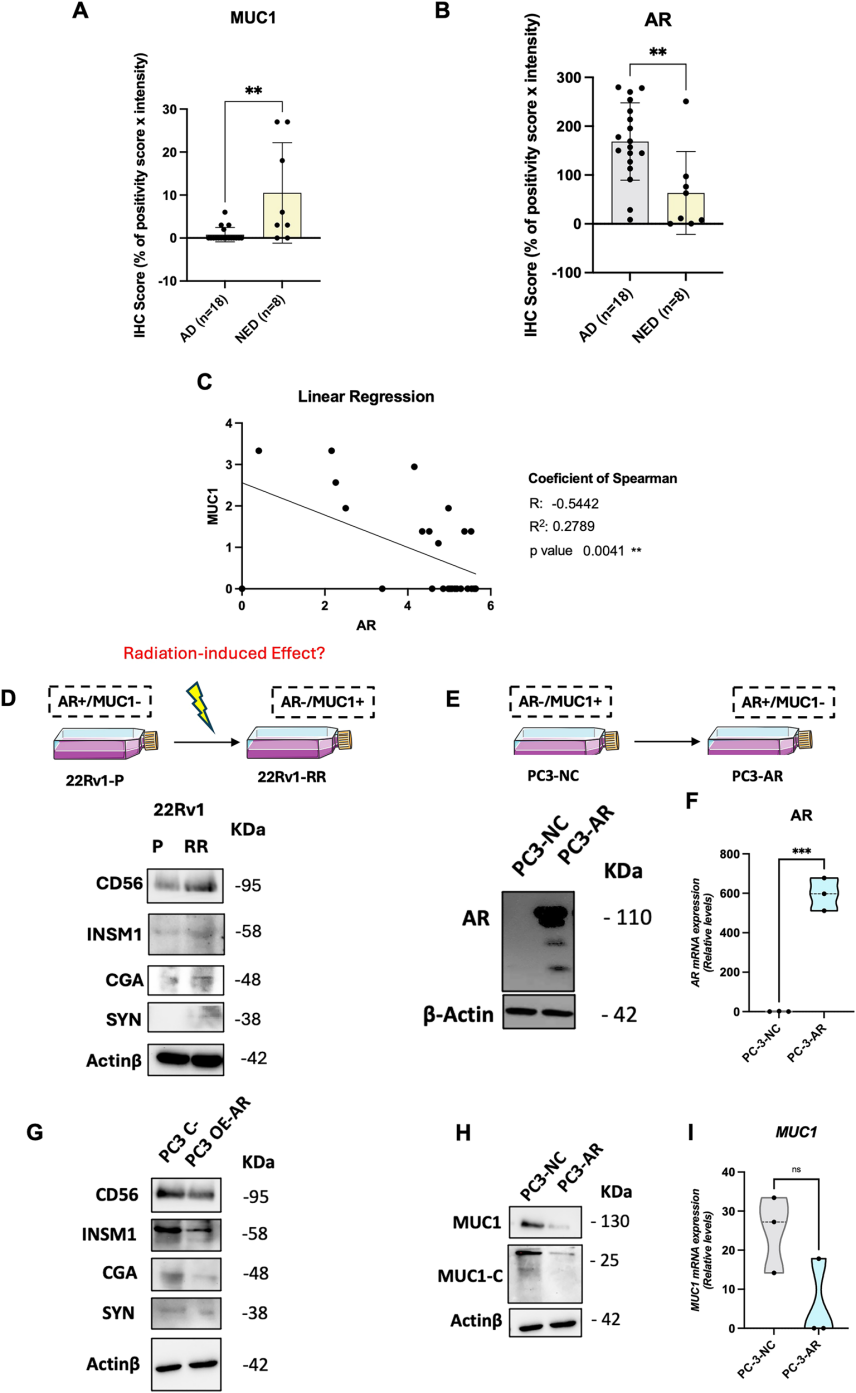
Mucin 1 (MUC1) is increased in NEPC and inversely correlated with androgen receptor (AR) expression. (**A**) AR and (**B**) MUC1 IHC score values comparing prostate adenocarcinomas (*n* = 18) with NEPC (*n* = 8) tissue samples, represented by scatter plot graphs with bars. **C** Scatter plot with a superimposed linear regression line. Each value was transformed using Ln (y + 1). The regression line shows a statistically significant negative correlation between AR and MUC1 (R² = −0.54, *p* < *0.01*). **D** Total protein levels of CD56 (95 kDa), INSM1 (58 kDa), CGA (48 kDa) and SYN (38 kDa) for 22Rv1-P (AR + /MUC1-) and RR (AR-/MUC1 + ) cells. **E** Total protein levels of AR (110 kDa), for PC3-NC (negative cell transfection control) and PC3-AR (overexpressing AR). **F** Relative mRNA expression levels of *AR* for PC3-NC and PC3-AR cells, normalized by *GUSB*gene expression levels (housekeeping). Results are presented as mean ± SD of at least 3 independent experiments. *** *p*-value < 0.001. **G** Total protein levels of CD56 (95 kDa), INSM1 (58 kDa), CGA (48 kDa) and SYN (38 kDa) for PC3-NC and PC3-AR cells. **H** Total protein levels of MUC1 (130 kDa) and MUC1-C (25 kDa) for PC3-NC and PC3-AR cells. β-actin (42 kDa) was used as loading control for all western blots (**D**–**G**) and the images were acquired by Chemidoc detection system (Biorad, Berkeley, California). **I** Relative mRNA expression levels of *MUC1* for PC3-NC and PC3-AR cells, normalized by *GUSB* gene expression levels (housekeeping). Results are presented as mean ± SD of at least three independent experiments. *ns*, not significant.

We first screened wild-type PCa cell lines for AR and MUC1 expressions (Supplementary figure 1A, B). PC3 and DU145, which are AR-negative PCa cell lines (Supplementary figure 1A), derived from castration resistant NE-like tumors, disclosed the highest MUC1 mRNA expression levels contrarily to the less aggressive cell lines C4-2 and 22Rv1 (Supplementary figure 1B). C4-2 and 22Rv1, which express high levels of AR, depicted reduced levels of MUC1 (Supplementary figure 1A, B). In PCa tissues, MUC1 protein was significantly overexpressed in advanced tumors with NED, compared to localized hormone-naïve prostate adenocarcinomas (Fig. 1A), while showing decreased AR immunoscore (Fig. 1B). A significant negative correlation between these two proteins was depicted [r = −0.54, *p* = 0.0041] (Fig. 1C)]. This data agrees with in-silico data from The Cancer Genome Atlas (TCGA) (Supplementary figure 1C). Additionally, in silico MUC1 expression positively correlates with classical NED markers, such as *NCAM1* (CD56), neuron specific enolase (*NSE)* and *CHGA* (chromogranin A) (supplementary figure 1D–F). Thus, we focused on elucidating the role of AR and MUC1 in a radioresistance model. Upon single-dose irradiation (SD-IR) with 2, 4, 6 and 8 Gy, the survival fraction (SF) of PC3 and DU145 cell lines (AR-/MUC1 + ) was significantly higher compared to C4-2 and 22Rv1 (AR + /MUC1-) (Supplementary figure 1G). Because MUC1 can mediate PCa lineage plasticity [12], we questioned whether AR-MUC1 dynamics might alter RT-induced NED. Firstly, we found that prolonged exposure to fractionated RT led to an increase of CD56, INSM1, CGA and Synaptophysin (Syn) in radioresistant (RR) cells – 22Rv1-RR - that we previously established [17] (Fig. 1D). Contrarily, in a gain-of-function model, we found that forced AR overexpression in PC3 cells (PC3-AR) (Fig. 1E, F) led to a decrease of NED-related markers (Fig. 1G). Moreover, PC3-AR cells showed significantly reduced MUC1 and MUC1-C protein and mRNA levels (Fig. 1 H, I). Together, these results suggest that MUC1, contrarily to AR, is overexpressed in CRPC NE-like cells, rendering them less responsive to RT.

### AR abrogation induces MUC1 expression upon prolonged RT exposure in PCa cells

**Fig. 2:**
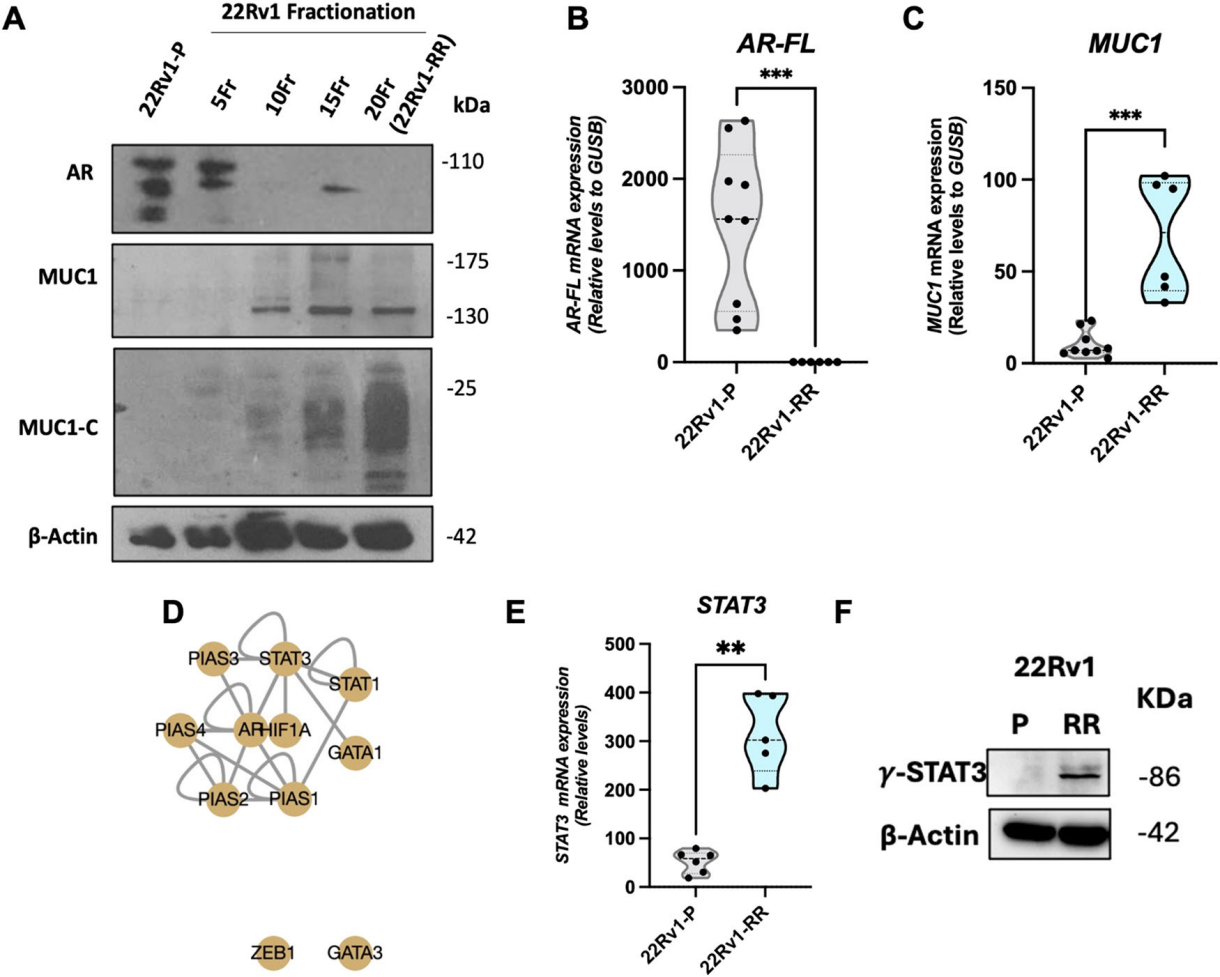
AR abrogation, MUC1-C de novo expression and STAT3 activation in 22Rv1-RR cells. **A** Total protein levels of AR full length-FL (110 kDa), MUC1 (130 kDa) and MUC1-C (25 kDa) in 22Rv1-P and -RR cells submitted to 5, 10, 15 and 20 fractions of 2.5 Gy. β-actin (42 kDa) was used as loading control. Relative mRNA expression levels of (**B**) *AR-FL* and (**C**) *MUC1* in 22Rv1-P and -RR cells. Results are presented as mean ± SD of at least three independent experiments. *GUSB* was used as reference gene for normalization. **D** Protein-protein interaction of MUC1 regulatory network. Data was retrieved from TRRUST v2 online platform (https://www.grnpedia.org/trrust/). **E** Relative mRNA expression levels of *STAT3* in 22Rv1-P and -RR cells. Results are presented as mean ± SD of at least three independent experiments. GUSB was used as reference gene for normalization. **F** Total protein levels of γ-STAT3 (86 kDa) in 22Rv1-P and -RR subpopulations. β-actin (42 kDa) was used as loading control. Western blot images (**A**, **F**) were acquired by Chemidoc detection system (Bio-Rad, USA).

Although the parental 22Rv1 cell lines (22Rv1-P) expressed high AR mRNA and protein levels, upon prolonged exposure to fractionation IR (FIR), both protein and transcript AR expression were dramatically lost (Fig. 2A, B), while MUC1/MUC1-C was expressed de novo (Fig. 2C). Indeed, AR protein levels were completely abrogated upon 10 fractions of IR (Fig. 2A). Next, we investigated possible MUC1 regulators by analyzing a protein-protein interaction network, using in silico platforms -TRRUST *version 2* (Fig. 2D). As anticipated, AR surfaced as a transcriptional suppressor of MUC1 expression. Conversely, STAT3, STAT1 and GATA3 stood out as relevant transcription factors that positively regulate MUC1 (Supplementary table 1). Remarkably, *STAT3* transcript levels (Fig. 2E) and its active phosphorylated form, ySTAT3, were significantly upregulated in 22Rv1-RR cells compared to parental cells (Fig. 2F). No significant differences were found, however, for *STAT1* and *GATA3* transcription levels (Supplementary figure 2). Remarkably, *STAT3* was that with more overlapping targeted genes with *AR*, comparing to the other two genes (Supplementary table 2).

### Epigenetic modulation of AR gene promoter in 22Rv1-RR cells

**Fig. 3:**
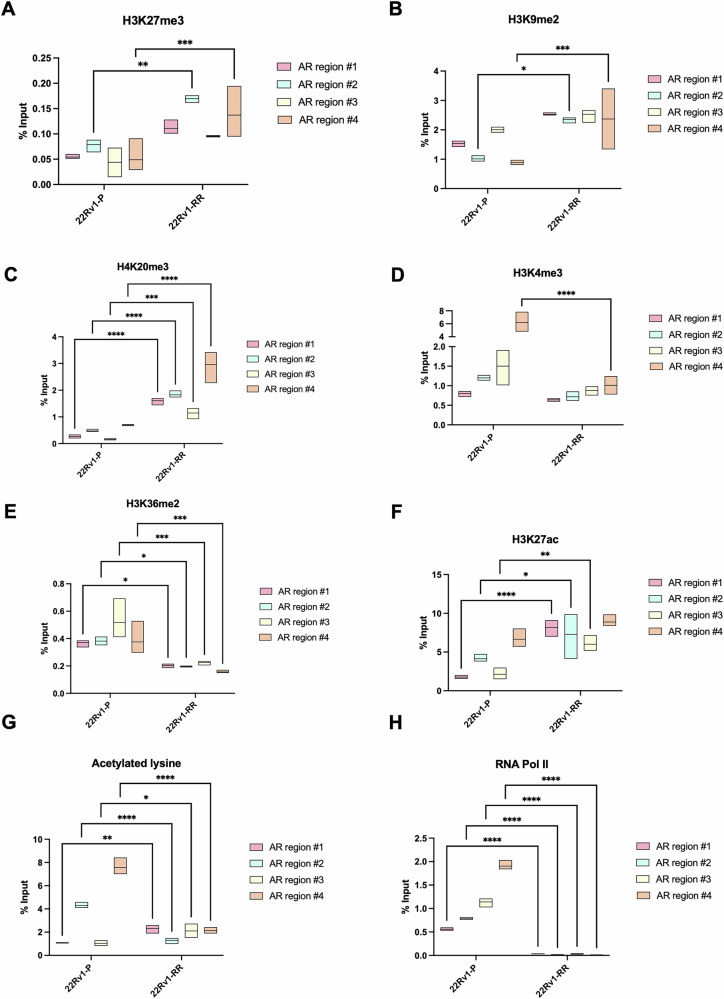
AR gene promoter epigenetic modulation via histone methylation dynamics in 22Rv1-RR cells. H3K27me3 (**A**), H3K9me2 (**B**), H4K20me3 (**C**), H3K4me3 (**D**), H3K36me2 (**E**), H3K27ac (**F**), global acetylated lysine (**G**) and RNA polymerase II (**H**) % input values at *AR* gene promoter in four different regions above transcription starting site (TSS) for 22Rv1-RR compared to 22Rv1-P cells. Graphs were represented by mean ± SD values. *Ns*, not significant, **p value < 0.01, ***p value < 0.001; ****p value < 0.0001.

To dissect the molecular mechanism by which RT triggers AR suppression to facilitate the expression of MUC1 and NED in PCa, we further focused on investigating the impact of epigenetic modifications on the transcriptional modulation of AR. The analysis of the AR promoter was performed across a ~ 3 kb region upstream of the transcription start site (TSS), divided into four distinct regions. We found a significant and impressive increase in the occupancy of histone repressive marks – H3K27me3, H3K9me2, and H4K20me3 - within the *AR* gene promoter region domain in 22Rv1-RR cells, compared with the parental cells (Fig. 3A–C). Conversely, constitutively active chromatin domains – H3K4me3, H3K36me2 and global lysine acetylation – were significantly downregulated at the same promoter region, except for H3K27ac, which increased *AR* occupancy (Fig. 3D–G). Importantly, the most pronounced changes were observed in region 4, located closest to the TSS ( ~1000 bp upstream). This pattern of epigenetic regulation of AR is consistent with our previous findings [11]. Specifically, the localized enrichment of the repressive mark H3K27me3 in the proximal promoter region, coupled with the global reduction in the activating marks (H3K4me3 and global histone acetylation), along with the absence of RNA Pol II binding (Fig. 3H) in 22Rv1-RR cells, provides strong evidence for AR transcriptional repression in this radioresistant cell model. Overall, we showed that *AR* is epigenetically regulated in PCa cells upon radioresistance.

### AR and STAT3 as transcriptional regulators of MUC1

**Fig. 4:**
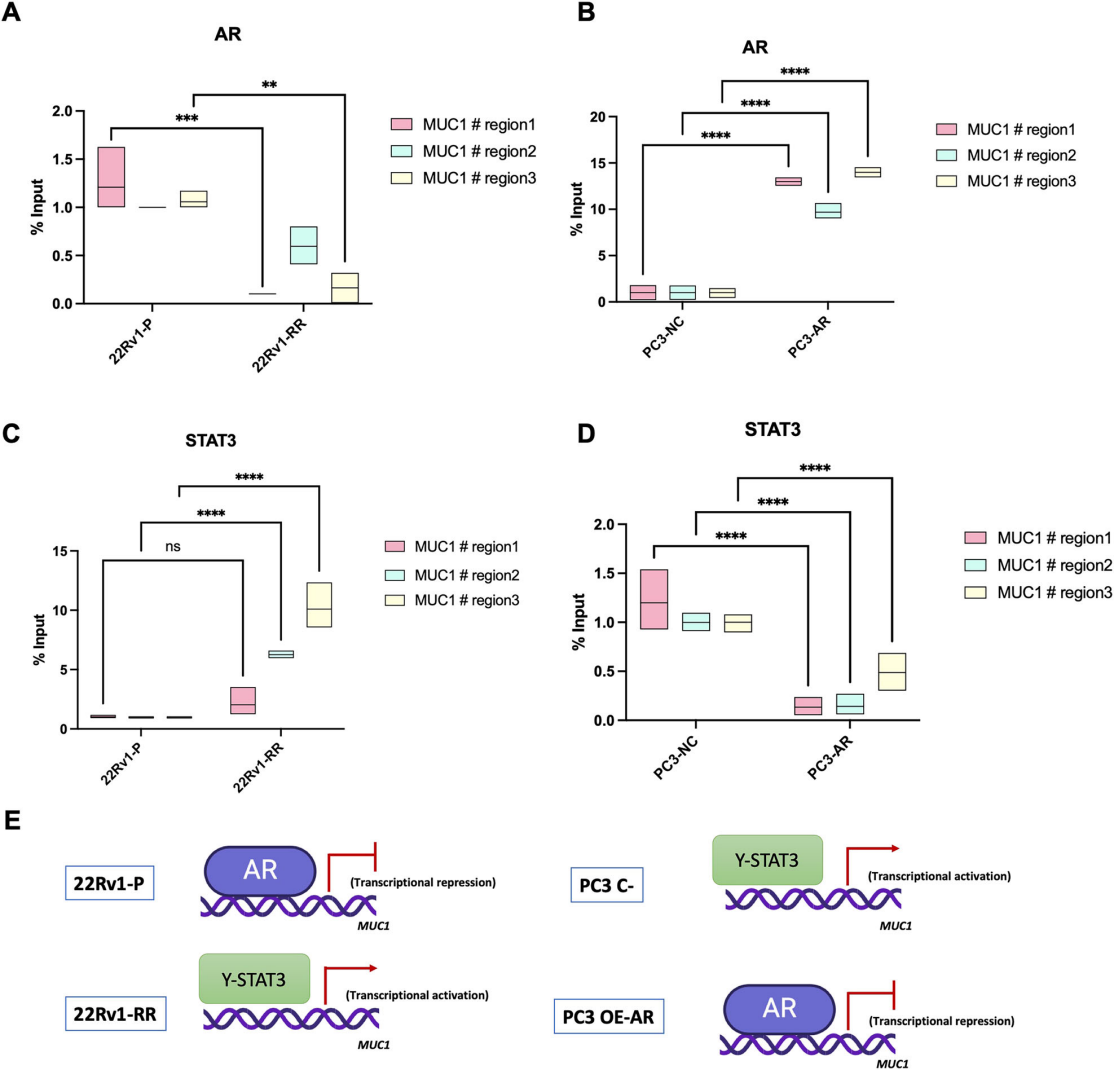
MUC1 gene promoter modulation by AR and STAT3. AR (**A**, **B**) and γ-STAT3 (**C**, **D**) % input values at *MUC1* gene promoter in three different regions above transcription starting site (TSS) for 22Rv1-RR (radioresistant) compared to 22Rv1-P (parental) cells and for PC3-NC and PC3-AR (AR overexpressing cells), respectively. Graphs were represented by mean ± standard deviation (SD) values. *p-value < 0.05, **p-value < 0.01; ****p-value < 0.0001. **E** Representative scheme of MUC1 gene promoter regulation by AR (Blue) and γ-STAT3 (green) binding. AR expression in 22Rv1-P and PC3-AR leads to MUC1 transcriptional repression, while AR downregulation in 22Rv1-RR and PC3-NC leads to MUC1 transcriptional activation.

Our data suggested that AR acts as a negative transcriptional regulator of MUC1 in radioresistant PCa cells, as confirmed elsewhere for AR-dependent and -independent cell lines [18]. Chromatin immunoprecipitation (ChIP) assay indicated that AR binds *MUC1* promoter region in 22Rv1 parental cells (Fig. 4A). Likewise, there was no binding in PC3 parental cells, which are AR-negative, but when AR was overexpressed, binding of AR to *MUC1* promoter region was depicted (Fig. 4B). We, then, hypothesized whether in the absence of AR, STAT3 might emerge as replacement, i.e., a positive regulator. Indeed, both in 22Rv1-RR and in PC3-NC cells, a significant positive binding of STAT3 to *MUC1* gene promoter was observed, compared to the 22Rv1-P and PC3-AR, respectively (Fig. 4C, D). The schematic panel in Fig. 4E illustrates the interaction of AR and STAT3 with *MUC1* gene promoter in both studied models – 22Rv1-P/RR and PC3-NC/AR. Furthermore, *STAT3* knockdown resulted in downregulation of both MUC1 and its cleaved subunit MUC1-C in 22Rv1-RR, PC3 and DU145 cell lines (Supplementary figure 3), indicating that *MUC1* expression is transcriptionally dependent on STAT3. In contrast, when AR is present, it appears to function as a transcriptional repressor, leading to MUC1 silencing.

### MUC1 silencing attenuates radioresistance of PCa cells

**Fig. 5:**
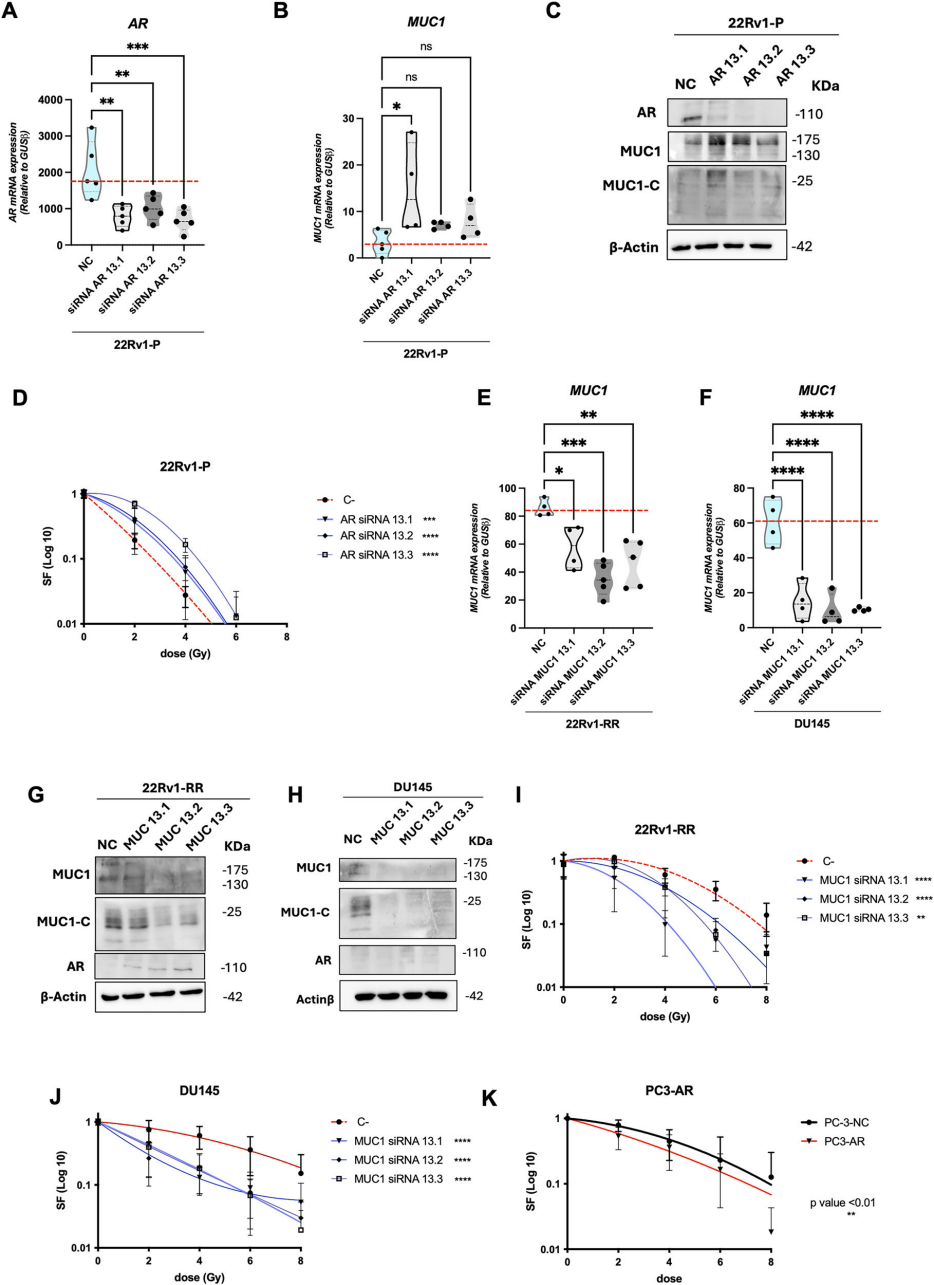
MUC1 Knockdown (KD) leads to the mitigation of radioresistant phenotype in 22Rv1-RR and DU145 cells. Relative mRNA expression levels of (**A**) *AR* and (**B**) *MUC1* in 22Rv1-P cells upon AR siRNA silencing with three different oligos (13.1 to 13.3) and the respective negative control. Results are presented as mean ± standard deviation (SD) of at least three independent experiments. *GUSB* was used as reference gene for normalization. **C** Total protein levels of AR (110 KDa), MUC1 (130-175 kDa) and MUC1-C (25 kDa) in 22Rv1-P cells upon AR siRNA silencing with three different oligos (13.1 to 13.3) and the respective negative control. β-actin (42 kDa) was used as loading control. Images were acquired by Chemidoc detection system (Bio-Rad, USA). **D** Cell survival fraction (SF) of 22Rv1-P upon AR siRNA silencing with three different oligos (13.1 to 13.3) and the respective negative control, represented through linear-quadratic model (LQ = (S = e ^– (xD + BD2)^)), ***, *p value* < *0.001* **** *p value* < *0.0001*. Relative mRNA expression levels of *MUC1* in 22Rv1-RR cells (**E**) and DU145 (**F**) cells upon MUC1 siRNA silencing with three different oligos (13.1 to 13.3) and the respective negative control. Results are presented as mean ± SD of at least three independent experiments. *GUSB* was used as reference gene for normalization. Total protein levels of AR (110 KDa), MUC1 (130–175 kDa) and MUC1-C (25 kDa) in 22Rv1-RR cells (**G**) and DU145 (**H**) cells upon MUC1 siRNA silencing with three different oligos (13.1–13.3) and the respective negative control. β-actin (42 kDa) was used as loading control. Images were acquired by Chemidoc detection system (Bio-Rad, USA). Cell survival fraction (SF) of 22Rv1-RR (**I**) and DU145 (**J**) cells upon MUC1 siRNA silencing with three different oligos (13.1 to 13.3) and the respective negative control, represented through linear-quadratic model (LQ = (S = e ^– (xD + BD2)^)), **, *p value* < *0.01* **** *p value* < *0.0001*. **K** Cell survival fraction (SF) of PC3 negative control (NC) and PC3-AR (AR overexpressing) cells, represented through linear-quadratic model (LQ = (S = e ^– (xD + BD2)^)), **, *p value* < *0.01*.

All previously described results suggest that prolonged exposure to RT may promote NED in PCa cells via alteration of the AR/MUC1/ STAT3 axis, leading to therapy resistance. To delve deeper into how response to RT depends on AR/MUC1 expression, we accomplished AR downregulation in 22Rv1-P cells, which closely mimics what we found to occur during prolonged RT exposure (Fig. 5A). Notably, AR knockdown (*AR*-*KD)* significantly increased MUC1/MUC1-C expression in 22Rv1-P cells, at least with the siRNA AR 13.1 oligo sequence (Fig. 5B, C). Furthermore, 22Rv1 cells with *AR* downregulation disclosed significantly increased clonogenicity when exposed to SD-IR from 0 to 8 Gy, which is indicative of a more resistant behavior (Fig. 5D). Next, we knocked down MUC1 expression (*MUC1-KD*) in both 22Rv1-RR and DU145 cell lines (Fig. 5E–H). Contrarily to AR knockdown, AR levels in *MUC1-KD* cells were not significantly altered (Fig. 5G, H and supplementary figure 4). Nonetheless, a slight increase in AR protein levels was observed in *MUC1-KD*-22Rv1-RR, but not in DU145 cells (Fig. 5G). Remarkably, both 22Rv1-RR- and DU145- *MUC1-KD* cells depicted significantly reduced clonogenicity upon SD-IR exposure (Fig. 5I, J). Moreover, PC3-AR cells, which express low levels of MUC1 (Fig. 1I, J), disclosed significant radiosensitivity compared to the negative control (Fig. 5K). Noteworthy, 22Rv1-*MUC1-KD* cells displayed a radiosensitivity profile closer to the original 22Rv1-P - *yellow arrow* (Supplementary figure 5). Altogether, these results suggest that *MUC1-KD* may enhance RT response even when AR levels are absent or residual. Indeed, MUC1, rather than AR, seems to act as a master radioresistant driver in our cell model.

### Mass spectrometry-based quantitative proteomics discloses significant differences between parental and RR cells

**Fig. 6:**
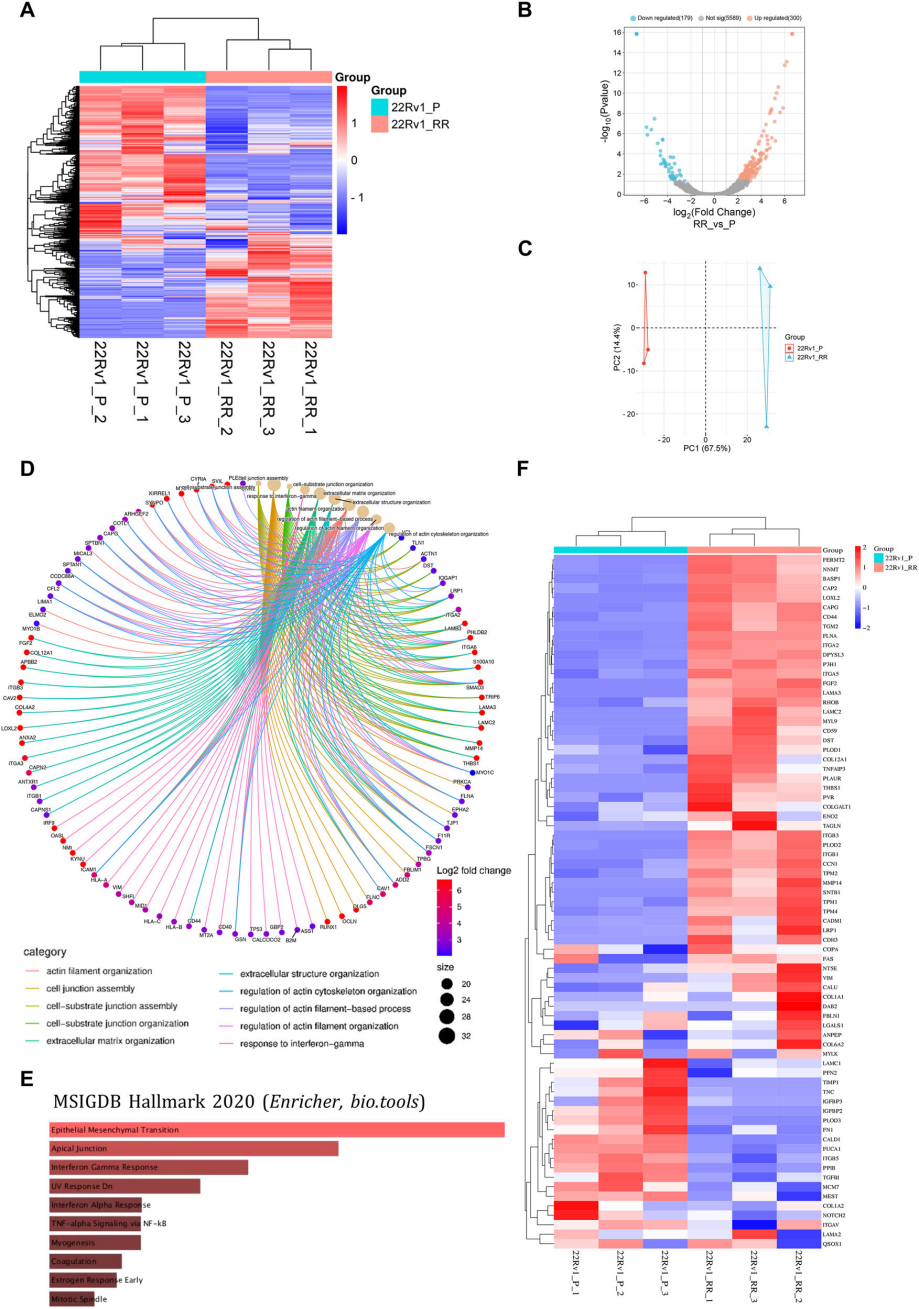
Identification of 22Rv1-RR cell traits and relevant signaling pathways by Mass spectrometry. **A** Heatmap representing the relative abundance of peptides identified in 22Rv1-P (parental) and -RR (radioresistant) samples using mass spectrometry (LC-MS) analysis. Rows represented the detected peptides and columns represent each analyzed samples, 22Rv1-P in light blue and 22Rv1-RR in light red, in independent triplicates. The color intensity of each square reflects the relative abundance of a specific proteins. The range is indicated by the scale bar with red indicating higher abundance, blue indicating lower abundance and white representing no abundance detected. **B** Volcano plot depicting log_2_ scale fold change versus negative log_10_-transformed *p-value* for all identified peptides. Light Red dots indicate peptides with statistically significant up-regulation (*p*-value < 0.05, fold change > 2), and light blue dots indicate peptides with statistically significant down-regulation (*p*-value < 0.05, fold change < −2). Sample RR (22Rv1-RR cells in triplicates) was compared with samples P (22Rv1-P cells in triplicates). **C** Scatter plot representing the distribution of 2Rv1-P and -RR samples in the first two principal components (PC1 and PC2). Each point represents a sample. The axes represented by PC1 and PC2 explain 67.5% and 14.4% of the total variance in the data, respectively. **D** Cytoscape network visualization (cNETplot) depicting the relationships between differentially expressed peptides following irradiation exposure in 22Rv1-RR cells. Nodes represent genes, and edges represent significant co-expression relationships between them. Node size corresponds to the absolute value of the log2 fold change. Node colour indicates the direction of fold change (red for up-regulated, blue for down-regulated). Edges are coloured based on the correlation coefficient between gene expression profiles. Colour legends are represented in the figure. Source: SRplot online platform. **E** Bar chart showing significantly enriched pathways in 22Rv1-RR cells identified through the proteomic analysis for the differential expressed peptides between 22Rv1-P (parental) and -RR (radioresistant) cells. Higher bar heights indicate stronger enrichment. Source: Online Enricher library. **F** Heatmap representing the relative abundance of peptides identified in 22Rv1-P (parental) and -RR (radioresistant) samples using Gene set enrichment analysis (GSEA), molecular signature database (MsigDB) for “hallmark of epithelial-mesenchymal-transition” Human gene set. Rows represented the detected peptides and columns represent each analyzed samples, 22Rv1-P in light blue and 22Rv1-RR in light red, in independent triplicates.

To unveil the most relevant altered molecular pathways linking MUC1/MUC1-C with a radioresistant-induced NED phenotype in PCa, mass spectrometry-based quantitative proteomics was performed in 22Rv1-P and RR cells. The results revealed significant differences in expression of a considerable number of peptides (Fig. 6A–C). Specifically, 300 proteins were significantly upregulated, whereas 179 were significantly downregulated in 22Rv1-RR cells compared to the parental cells (Fig. 6B). As anticipated, classical NED markers such as CGA (*CHGA*) and enolase 2 (ENO2) were upregulated in the RR group (Supplementary figure 6A). Conversely, classical prostate gland markers that are lost in NEPC, such as AR and PSA (*KLK3*), were found downregulated (Supplementary figure 6A). Interestingly, CD49f and KRT14, which are commonly associated with basal cell-like traits, were found upregulated in the RR model, also suggesting increased cell aggressiveness with the loss of luminal cell identity (Supplementary figure 6A).

The transition to invasive NED PCa cells upon prolonged exposure to IR was also associated with progressive changes in the structure and morphology of 22Rv1-RR cells (Supplementary figure 7A, B). Indeed, the established RR cells disclose significant morphometric changes in comparison with the parental fraction. Specifically, 22Rv1-RR cells are larger, with increased circularity, roundness, and solidity, while displaying reduced skewness (Supplementary figure 7C–H), further supporting that RT-induced AR-STAT3-MUC1-MUC1-C axis drives cell plasticity and aggressiveness in advanced PCa.

Overall, gene ontology analysis revealed significant differences in biological processes related to the structure and organization of actin filaments, cell-cell junction, focal adhesion, and cytoskeleton organization and response to interferon gamma (Fig. 6D). In particular, analysis of Kyoto Encyclopaedia of Genes and Genomes (KEGG) pathways emphasized gene sets related to focal adhesion and cell adhesion molecules, highlighting those that were up (red)- or down (green)-regulated in 22Rv1-RR group comparing with 22Rv1-P samples (Supplementary figure 6B, C). Moreover, upon analysing the data using Human MSigDB Hallmark Gene Set we found that epithelial mesenchymal transition (EMT) was the most significant molecular signaling pathway altered in our RR model, followed by apical junction, interferon gamma and response to UV radiation (Fig. 6E). Subsequently, we confirmed that a high number of proteins involved in EMT pathway were upregulated in 22Rv1-RR replicates compared with 22Rv1-P (Fig. 6F). Among them, p-cadherin (CDH3) and vimentin (VIM), both constituting relevant peptides involved in EMT, were prominently and significantly upregulated in 22Rv1-RR cells (Supplementary figure 6D). Using WB, we further confirmed VIM and PCAD to be considerably upregulated in 22Rv1-RR cells compared to parental fraction (Supplementary figure 6E). Conversely, VIM expression was decreased in PC3-AR cells compared with the negative control (Supplementary figure 6F), whereas PCAD was absent in PC3 cells (*data not shown*).

Interestingly, analysis of the top enriched molecules revealed that 22Rv1-RR were similar to DU145 cells (Supplementary figure 6G), although significantly different from the original 22Rv1 cell line, as well as from normal adult prostate tissue (Supplementary figure 8A, B). Likewise, many AR network-related molecules were downregulated in 22Rv1-RR compared to 22Rv1-P cells (supplementary figure 8C).

In summary, 22Rv1-RR cells exhibited a more mesenchymal phenotype characterized by traits associated with aggressiveness, including basal cell-like features, stemness, and neuroendocrine differentiation. These changes were accompanied by a reduction in AR signalling.

### AR/MUC1 loop disruption leads to abnormal cancer cell migration and invasion

**Fig. 7:**
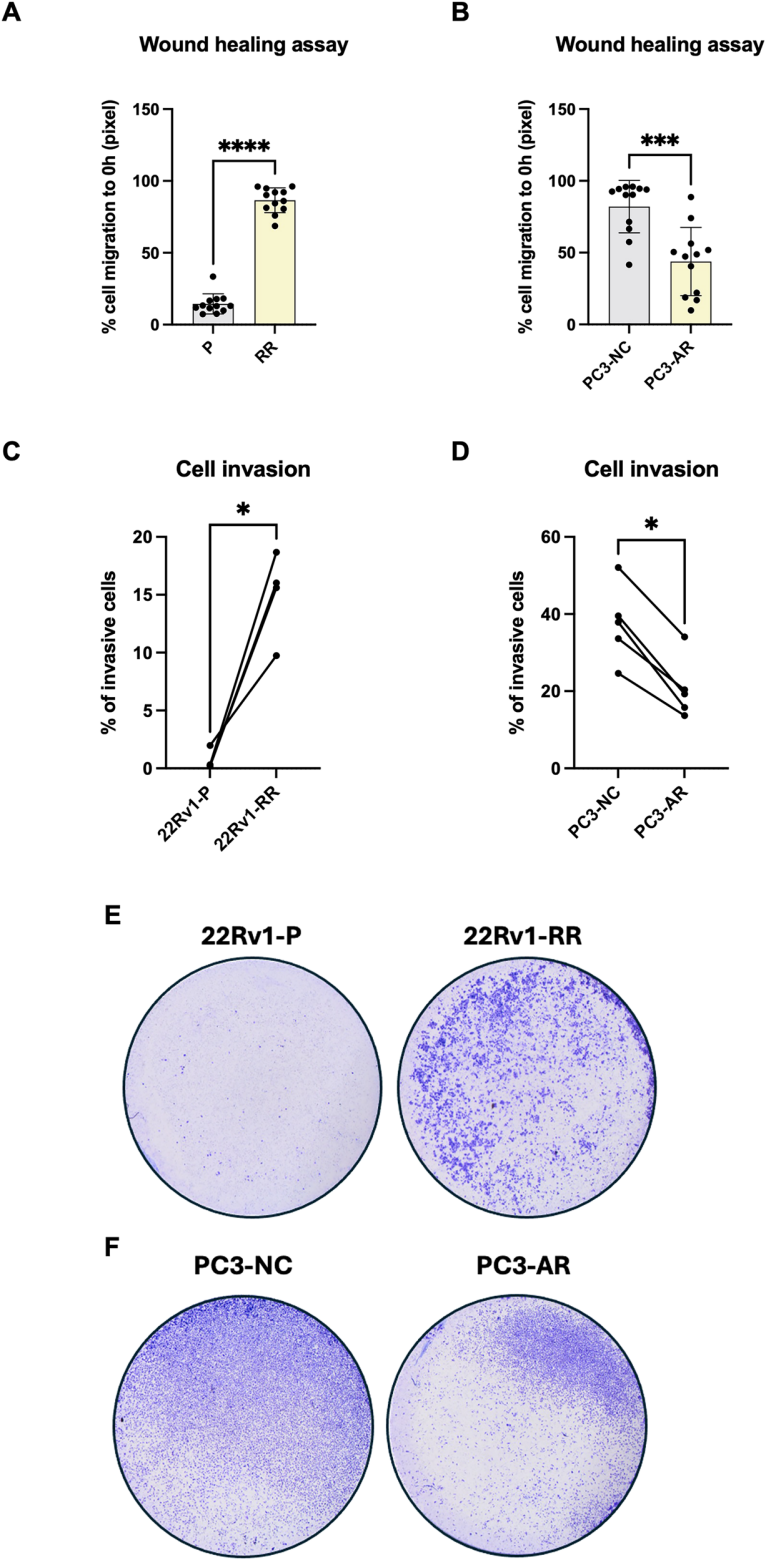
MUC1 overexpression led to higher PCa cell migration and invasion. Cell migration differences between 22Rv1-P and -RR cells and between PC3 negative control (PC3-NC) and AR expressing (PC3-AR) cells, **A** and **B**, respectively, during 24 h after wound formation. Results are represented by mean ± standard deviation (SD) in scatter plot with bars. yy axis represent the % of cell migration regarding the control timepoint of wound formation (0 h). *** *p* value < 0.001, **** *p* value < 0.0001. **C**, **D** Global changes in the percentage of invasive cells in transwell inserts and the respective plots with dots and lines confirming the evolution of each individual replicate between 22Rv1-P and -RR cells and between PC3 negative control (PC3-NC) and AR expressing (PC3-AR) cells, respectively. * *p* value < 0.05. **E**, **F** Representative images of cell invasion membranes in 22Rv1-P and RR (upper) and PC3-NC and PC3-AR (down), taken by a stereomicroscope Olympus S2X16 at 7x amplification. Violet dots are invasive cells.

Next, we performed migration and invasion assays using radioresistant and AR overexpressing cells. A more pronounced *wound healing* ability was observed in 22Rv1-RR cells compared to the parental fraction, with statistically significant differences (Fig. 7A). In PC3-AR cells, overexpressing AR and low MUC1, the opposite was observed, with a significant decrease in cell migration (Fig. 7B). Furthermore, the RR fraction (AR-/MUC1 + ) showed significantly enhanced cell invasion capability (Fig. 7C, E). Conversely, in PC3-AR cells (AR + /MUC1-), invasion capacity decreased by more than half compared with control cells (Fig. 7D, F), supporting a role for MUC1 in orchestrating an aggressive phenotype in PCa cells.

## Discussion and Conclusions

PCa remains a major health concern, having a significant impact on patient survival and quality of life [19]. Intra-tumoral heterogeneity, as well as divergent molecular and biological signatures are puzzling features of PCa, contributing to primary treatment failure and consequent disease progression [20, 21]. The versatility of AR throughout disease progression and its consequent depletion in more aggressive forms, like NEPC, poses significant challenges, especially considering that most primary therapies target this receptor, aiming to reduce prostate-specific tumor proliferation [22, 23]. Exploring the effects of androgen signaling suppression and further identifying new therapeutic targets for those patients at risk, may improve the effectiveness of first-line cell-killing therapies, such as RT. Earlier studies suggested that ADT and/or radiotherapy might drive treatment-induced NED, resulting in lack of response to therapy [4, 24, 25]. The phenomenon of tNED, stemming from primary therapy, may also be associated with the molecular and functional reprogramming of the tumor cells. The underlying mechanisms, however, have not been completely elucidated, thus far. Herein, the generation of a radioresistant in vitro model – 22Rv1-RR cells – allowed us to uncover a previously unrecognized role for MUC1-C oncoprotein in driving PCa radioresistance, disclosing evidence of the existence of a regulatory axis involving AR and MUC1 which drives PCa lineage switching towards NE-like characteristics.

We found that 22Rv1-RR cells exhibited de novo expression of MUC1/MUC1-C coupled with suppression of AR axis signaling. Besides 22Rv1-RR cells, we also showed that PC3 and DU145, which are metastatic castration-resistant PCa cells, constitutively express MUC1/MUC1-C oncoprotein, while expressing only residual to undetectable levels of AR [12]. Early findings demonstrated that those cells lines harbor NED traits, evidenced through expression of well-established NE cell markers [26]. Interestingly, MUC1 has been recognized as a driver of PCa lineage plasticity and NED [12], with MUC1 expression associating with NEPC score, in in silico datasets from TCGA [12]. Here, in a cohort of localized prostate adenocarcinomas and NEPC tissues, we confirmed AR downregulation and MUC1 overexpression, with a negative correlation. Moreover, we observed that prolonged irradiation exposure led to AR suppression, MUC1 upregulation and acquisition of NED features in PCa cells. Indeed, the increase of MUC1 expression in 22Rv1-RR cells was accompanied by increased NE markers expression, including CD56, INSM1, CGA and SYP. Interestingly, NE markers expression decreased when AR was overexpressed in PC3 cells, with a concomitant reduction of MUC1 expression, further emphasizing the negative correlation between the expression of those two proteins – AR and MUC1.

Previous studies substantiated the crosstalk between MUC1 and STAT3 as a downstream target in an auto-inductive regulatory loop [27–29]. In our study, we further demonstrated that AR functions as a transcriptional regulator of MUC1. Indeed, in the absence of AR, a significant occupancy of STAT3 at the *MUC1* promoter in both 22Rv1-RR and PC3 cells, which are AR-negative cell lines, was disclosed. Interestingly, MUC1-C and JAK-1 were reported as intermediators of STAT3 phosphorylation [28]. In the same vein, we found an upregulation of γ-STAT3 in 22Rv1-RR compared to the parental cells. Overall, these findings substantiate the existence of a regulatory mechanism involved in PCa radioresistance, in which epigenetic silencing of AR leads to MUC1 expression via STAT3 activation, which in turn drives tumor cell reprogramming towards a more mesenchymal phenotype, with stemness and NED traits.

Remarkably, mass spectrometry data revealed differential expression patterns between the parental lineage and the RR fraction. Among them, cell-cell adhesion and dynamics on actin filaments were the most common cellular alterations found in 22Rv1-RR cells, which are suggestive of evolution to an EMT phenotype, a recognized hallmark of cancer progression [30]. Moreover, the increased roundness observed in 22Rv1-RR cells has been considered to indicate a superior ability to invade the extracellular matrix [30], which is aligned with the observed increase in cell migration and invasion capacity of 22Rv1-RR. Interestingly, NE (CHGA and ENO2) and basal (cytokeratin 14, KRT14 and CD49f) cell markers were also found upregulated in 22Rv1-RR compared to 22Rv1-P cells, according to the proteomic analysis. It has been previously documented that CD49f positive cell populations overlapped with genes expressed in basal, stem and neuroendocrine cells and were associated with EMT, thus influencing cell invasion and migration [31]. Our results further confirm those observations and provide an additional explanation for the biological aggressiveness disclosed by 22Rv1-RR cells.

In conclusion, we demonstrated in this study that prolonged radiotherapy entails loss of AR and upregulation of MUC1 in a PCa subpopulation, driving NED and fostering EMT, materialized in a phenotypic and morphological switch, which ultimately translates into resistance to RT. In the past, attempts to restore AR expression through epigenetic inhibition have been unsuccessful due to tumor cell plasticity [11]. Herein, we show that MUC1 knockdown in 22Rv1-RR and DU145 cells significantly boosts RT response, even without restored AR expression, indicating the MUC1, per se, has an important role in driving radioresistance, independently of the MUC1/AR axis. Interestingly, MUC1 was previously associated with radioresistance in other models, but not in PCa [13–15]. Despite the well-known evidence that AR is a preponderant factor for PCa progression, in a radioresistance scenario in which more aggressive phenotypes emerge and typically lose AR expression, other targets should be considered for alternative therapeutic strategies, among which MUC1 cell surface oncoprotein seems of major interest.

## Materials and Methods

### Cell culture

PCa cell lines, including hormone-sensitive C4-2 and 22Rv1 and hormone-insensitive PC-3 and DU145, were selected for this study. Furthermore, 22Rv1-RR cells were used as a model of radioresistance [17]. Irradiated cells were collected for further protein assessment in each 5 IR fractions of 2.5 Gy. All cells were cultured with RPMI 1640 supplemented with 10% of fetal bovine serum (FBS), 100 IU/mL penicillin and 100 µg/mL streptomycin. Optimal cell culturing was maintained at 37 °C in a humidifier incubator with 5% CO_2_. Cells were irradiated as previously described for in vitro assays [17, 32]. Cells were periodically tested for Mycoplasma contamination using MyTaq HS Red PCR Mycoplasma Detection Set (Bioline, Meridian Bioscience, London, UK). Cell line authentication was performed by Genomics Scientific Platform at i3S – Institute for Research & Innovation in Health, University of Porto, Portugal. Complete identity of this cell line was confirmed by genotyping.

### PC3-*AR* overexpressing cells

Cell transfection was carried out by pEZ-Lv105 (GeneCopoeiaTM, Rockville, MD, USA) using FuGENE® HD Transfection Reagent (Promega, Madison, WI, USA), following manufacturer’s recommendations. In vitro cell transfection was performed when the cells had reached at least 30–50% of confluence. 2 μg of oligo molecules were diluted in Opti-MEM^TM^ medium (GIBCO®) to a final volume of 100 μL. Additionally, 4 μL of transfection reagent were added in a proportion of 2:1 FuGENE® HD Transfection Reagent:DNA ratio. Transfection of the cells with scramble DNA oligos was performed, serving as negative controls (NC). After 48 h of transfection, stable clones with the vector were selected with Puromycin dihydrochloride (cat. 631306, Clontech Laboratories Inc.) at a cytotoxic tested concentration of 0.5 μg/mL. AR overexpression was confirmed by western blot and real time quantitative polymerase chain reaction (RT-qPCR).

### siRNA transfection for *MUC-1* and *AR* gene silencing

Trifecta dicer subtracts containing three different siRNA sequences were used to knockdown MUC1 (hs.Ri.MUC1.13.1 to hs.Ri.MUC1.13.3) or AR (hs.Ri.AR.13.1 to hs.Ri.AR.13.3) (Integrated DNA technologies (IDT), USA). Also, a negative control siRNA sequence (DsiRNA, 1nmol), was used (Integrated DNA technologies (IDT)). Silencing of *MUC1* and *AR* gene expression by in vitro siRNA transfection was performed with Lipofectamine® 3000 reagent (Invitrogen, USA), according to manufacturer instructions. Dye labeled reagent was used for assessing cell transfection success, as confirmed by the red dots present inside the cells (supplementary figure 9). Gene silencing was confirmed for every experiment after 48 h of siRNA transfection, a reference time point for cell irradiation (0 h).

### Clonogenicity assay

PCa transfected cells (control, knockdown or overexpressing cells) were used for colony formation assay (CFA). Briefly, cells were plated in 24-well plates 48 h before IR exposure, at an adjusted density, upon siRNA transfection. Then, 1000 or 2000 cells per well were used for DU145 and PC3 or for 22Rv1-P and -RR, respectively, for colony formation in 6-well plates.

All cells were maintained in low densities for 7 days after ionization exposure in a range of 0, 2, 4, 6 and 8 Gy. Following colony formation, cells were stained using 1% Crystal Violet reagent in 20% methanol solution. Each colony was considered for the count if composed of at least 50 cells. Radiobiological cell survival curves were constructed using linear quadratic (LQ) model [S = e – (αD + βD2)], as previously described [17]. The plating efficiency (PE) of each independent experiment was calculated according to the initial number of cells seeded. PE = % (number of colonies counted in the control/number of cells plated). Then, the survival fraction was calculated taking into account the PE [SF=number of colonies counted/(number of cells plated*(PE/100))]. SF values were introduced in GraphPad Prism software version 9.1.1 to assess cell survival curves through LQ model.

### Protein extraction, SDS-PAGE Western Blot

Total protein extraction was performed as previously described [32]. Protein quantification was made by colorimetric detection using PierceTM BCA Protein Assay kit, according to manufacturer instructions. Western blot was performed using 50μg of total protein extract. Anti-AR monoclonal antibody (AR 441, MA5-13426, Invitrogen, USA), anti-MUC1 antibody (VU4H, sc-7313, Santacruz Biotechnology), and anti-MUC1-C (D5K9I, Cell Signalling technology), were used. NED-related protein expression was evaluated using monoclonal antibodies against CHGA (DAK-A3, DAKO), INSM1 (sc-271408, Santacruz Biotechnology) and CD56 (CD56-504-L-CE, Leica). All primary antibodies were diluted in TBS-T solution with 5% of bovine serum albumin (BSA) at the manufacturer recommended dilutions and inoculated with nitrocellulose membranes overnight at 4 °C. Anti-β-Actin antibody (A1978, Sigma Aldrich) was used as loading normalizer. All the original, uncropped western blot images are compiled in Supplementary figure 10.

### In silico studies and online databases

TCGA PanCancer database for prostate adenocarcinoma, derived from a large cohort of 488 PCa patients, was used to assess MUC1, AR and NED-related genes mRNA expression levels. Correlations were evaluated by Pearson or Spearmen statistical analysis. Data were downloaded from cBioPortal “For Cancer Genomics” online platform (https://www.cbioportal.org). TRRUST v2: an expanded reference database of human and mouse transcriptional regulatory interactions. Nucleic Acids Research 26 Oct, 2017 (https://www.grnpedia.org/trrust/) was used to unveil transcriptional regulatory networks of *MUC1* gene and to identify putative transcriptional repressors.

### Chromatin immunoprecipitation-ChIP (RT-qPCR)

ChIP- qRT-PCR was performed as previous detailed [17]. Specifically, to evaluate the binding affinity of histone modifications on *AR* gene promoter region, four pairs of primer sequences were used: P1, Forward: 5’AAATTTGGTGAGTGCTGGCCT 3’; Reverse: 5’AGGACCCCTGCTTCCTGAATA 3’; P2, Forward: 5’GGAGCTATTCAGGAAGCAGGG 3’; Reverse: 5’ TGGCTTTGGAGAAACAAGTGC 3’; P3, Forward: 5’CTCCAAAGCCACTAGGCAGG 3’; Reverse: 5’ GGTGGAGAGCAAATGCAACA 3’; P4, Forward: 5’TGTTGCATTTGCTCTCCACCT 3’; Reverse: 5’CCTTTTTCCCTCTGTCGCCT 3’. Primer annealing temperature was 62 °C for all the sequences, except for AR-P1 which was set at 60 °C. Furthermore, three different sequences over *MUC1* transcription starting site (TSS) were interrogated for AR and STAT3 binding – P1: Forward: 5’ TTGTCACCTGTCACCTGCTC 3’; Reverse: 5’ GGGCAGAACAGATTCAGGCA 3’; P2: Forward: 5’ AGCTGGAGAACAAACGGGTA 3’; Reverse: 5’ CCTCCCCTACCTCCTACCTCT 3’; P3: Forward: 5’ CTAGCTGGCTTTGTTCCCCA 3’; Reverse 5’ CCTTTCACCAACCACTCCCT 3’. Primer annealing temperature was 60 °C for P1 and P2 and 64 °C for P3.

### Total RNA isolation, cDNA synthesis and RT-qPCR

Total RNA extracts were obtained from all cell lines at each independent condition, using Trizol reagent-based extraction method, followed by cDNA synthesis of 1000 ng of RNA using RevertAid RT kit (Thermo Fisher Scientific Inc., Waltham, MA, USA), according to manufacturer’s instructions. Relative gene transcription levels were calculated using *GUSB* as housekeeping gene. Primer sequences and the respective optimized annealing temperature are listed in Supplementary table 3.

### Immunohistochemistry in formalin-fixed paraffin embedded (FFPE) tissues

Anti-AR monoclonal antibody (AR 441, MA5- 13426, Invitrogen) and anti-MUC1 (VU4H, sc-7313, Santacruz Biotechnology) antibodies were used to assess protein expression by immunohistochemistry (IHC), using a NovoLinkTM Max Polymer Detection System (Leica Biosystems, Germany). Briefly, antigen retrieval was performed in a microwave oven at 800 W for 20 minutes in citrate 1x for MUC1, or in 95–100 °C water-bath in EDTA 1x for 30 minutes for AR, followed by 10 min cooling at room temperature. Next, tissue slides were incubated with 3% H_2_O_2_ in methanol (GRiSP, Portugal) solution for 10 min, at room temperature. Additionally, the slides were blocked with horse serum (Vector Laboratories, USA) diluted at 1:50, for 20 min, and subsequently incubated overnight with the primary antibody, at 1:250 for AR and 1:300 for MUC1. Staining analysis was performed blinded regarding the groups. AR nuclear staining was evaluated using a quantitative method from GenASIS software (Applied Spectral Imaging, ASI), considering the percentage of positive cells and the intensity of immunostaining, providing a continuous variable: IHC score [1 × (% of cells stained weakly) + 2 × (% of cells stained intermediately) + 3 × (% of cells stained strongly)]. MUC1 staining was assessed by a dedicated uropathologist. Staining intensity was categorized between 0 and 3 (0 = negative, 1 = weak, 2 = moderate and 3 = strong). Then, the percentage of positive cells were categorized as <5% followed by 10% interval categories. Extension score was defined from 0 to 9, according to the category of positivity percentage. Lastly, IHC score was calculated by multiplying intensity with extension scores. IHC images were acquired using an Olympus BX41 microscope equipped with a digital camera (Olympus U-TV0.63XC) and CellSens software (version V0116, Olympus).

### Cell migration (“*wound healing*”) assay

Briefly, 6 × 10^5^ cells were seeded into 6-well plates in 2 mL of complete RPMI culture medium and grown until confluence was reached. For PC3 cell lines, PC3 NC condition healed the wound after 20 h. Instead, 22Rv1-RR cells reached the same effect only after completing 24 h. All experiments were photographed using Olympus IX51 inverted microscope equipped with Olympus XM10 Digital Camera System. The relative migration distance was computed as relative migration distance (%) = (A – B) / C × 100, where A represents the initial width of the cell wound formation, B represents the width of the cell wound after a 20 h or 24 h, for PC3 and 22Rv1 cells, respectively, and C represents the mean width of the initial cell wound. beWound—Cell Migration Tool (Version 1.5) was used to conduct the analysis of relative migration distances, based on results from at least three independent experiments.

### Transwell in vitro cell invasion assay

24-well BD Biocoat Matrigel Invasion Chambers (BD Biosciences) were used to address PCa cell invasion capabilities. Two days after cells platting, non-invading cells were removed by cotton swab and the invading cells were fixed with cold methanol during 20 min, followed by staining with 1% Cristal Violet diluted in 20% methanol. Invaded membranes were captured using Olympus SZx16 stereomicroscope [16x], and the percentage of invading cells were computed using Image J software (version 1.41; NIH). At least three independent experiments were performed.

### Mass spectrometry-based quantitative proteomic analysis

Using 22Rv1-P and RR cell line protein extracts, a comprehensive proteomic analysis was performed. Briefly, around 30 µg of total protein per sample was enzymatically digested with trypsin/LysC. Protein identification and quantification was then performed by nanoLC-MS/MS using a Vanquish neoliquid chromatography system coupled to an Eclipse Tribrid QuadrupoleIon trap Orbitrap mass spectrometer together with a High-field asymmetric waveform ion mobility spectrometry – FAIMS equipment (Thermo Scientific). The raw data was processed using Proteome Discoverer software (Thermo Scientific) and searched against the UniProt database for the *Homo sapiens* proteome. A common protein contaminant list search was performed for further discrimination. The mass spectrometry proteomics data have been deposited to the ProteomeXchange Consortium with the dataset identifier PXD012345.

To gain insights into the statistically significant under- or over-expressed proteins in 22Rv1-RR cells across multiple biological sample sources, exploratory and protein label free quantification - LFQ analyses were performed. Functional Enrichment analysis, including Over-Representation Analysis and Gene Set Enrichment Analysis were also carried out to further understand the functional roles of identified proteins. Thus, protein data was queried to Gene Ontology and pathway functional databases, including KEGG and Reactome.

### Statistics

All data were analyzed using GraphPad Prism software version 9.1.1. Normality tests (Shapiro-Wilk) were applied to all datasets to define the adequate subsequent statistical analysis: parametric tests (two-way ANOVA or Student’s t-test) for data following a normal distribution, and non-parametric tests (Mann-Whitney or Kruskal–Wallis, to compare two or more groups, respectively) for non-normal data distribution. When ANOVA test was used, Brown-Forsythe and Welch tests were applied assuming no equal standard deviation (SD). Clonogenicity cell survival curves were constructed based on the linear quadratic model SF=e – (αD + βD2) and global differences among curves were computed using least squares regression fitting model and the comparison method of extra sum-of-squares F test, selecting both alpha and beta parameters of the equation. Non-parametric Spearman correlation was performed between AR and MUC1 IHC-variables, previously transformed by the function of Y= Ln (1 + Y). Simple linear regression was computed to find the linear relationship between the two described variables.

## Supplementary information

Supplementary figure legends

Supplementary figure 1

Supplementary figure 2

Supplementary figure 3

supplementary figure 4

supplementary figure 5

supplementary figure 6

supplementary figure 7

supplementary figure 8

supplementary figure 9

supplementary figure 10

Original data

Supplementary tables

## Data Availability

The authors confirm that the data supporting the findings of this study are available within the article and in Supplementary material file. The mass spectrometry proteomics data have been deposited to the ProteomeXchange Consortium with the dataset identifier PXD012345. Other raw data that support the findings of this study are available from the corresponding author, upon reasonable request.
