# Supplementary material for: Decoding MUC1 and AR axis in a radiation-induced neuroendocrine prostate cancer cell-subpopulation unveils novel therapeutic targets
Source: Cell Death Discov. 2025 Jul 3;11:306. doi: 10.1038/s41420-025-02597-4 (PMC12229644; doi:10.1038/s41420-025-02597-4)
Supplement: Supplementary file 1 — Supplementary figure legends [file 41420_2025_2597_MOESM1_ESM.docx]

***Macedo-Silva et al.* *Supplementary figure captions***

**Supplementary fig. 1. AR, MUC1 and neuroendocrine markers expression in PCa cell lines. Ionizing-radiation response among PCa cells.** A) and B) mRNA relative expression levels of **A)** *AR* and **B)** *MUC1*, normalized by *GUSB* gene expression levels (housekeeping), in C4-2, 22Rv1, PC3 and DU145 wild-type cell lines. Results are presented as mean ± SD of at least 3 independent experiments. C), D), E), F) Correlation analysis between *MUC1* and *AR*, *NCAM1*, *NSE* and *CHGA*, respectively. Spearmen correlation coefficient was used as the statistical method to evaluate the significance. Data was retrieved from GEPIA Tang, Z. et al. (2017) GEPIA: a web server for cancer and normal gene expression profiling and interactive analyses. The graphs included raw data from TCGA and GTEX databases including both normal and tumor samples. G) Survival fraction of C4-2, 22Rv1, PC3 and DU145 wild-type cells upon 0-8Gy single dose irradiation.

**Supplementary fig. 2. *STAT1* and *GATA3* expression in 22Rv1-RR cells.** mRNA relative expression levels of **A)** *STAT1* and **B)** *GATA3*, normalized by *GUSB* gene expression levels (housekeeping), in 22Rv1-RR cells compared to the parental ones (22Rv1-P). Results are presented as mean ± SD of at least 3 independent experiments. ns, not significant.

**Supplementary fig. 3. *STAT3 knockdown influences MUC1* expression.** Relative mRNA expression levels of *STAT3* (A, B and C) and *MUC1* (D, E and F) for 22Rv1-RR, PC3 and DU145 cells, respectively, upon STAT3 siRNA silencing with three different oligos and the respective negative control. Results are presented as mean ± standard deviation (SD) of at least three independent experiments. *GUSB* was used as reference gene for normalization. Total protein levels of γ-STAT3 (86 KDa), MUC1 (130-175kDa) and MUC1-C (25kDa) in 22Rv1-RR (G), PC3 (H) and DU145 (I) cells upon STAT3 siRNA silencing with three different oligos and the respective negative control. β-actin (42kDa) was used as loading control. Images were acquired by Chemidoc detection system (Bio-Rad, USA).

**Supplementary fig. 4. *AR* expression in *MUC1* knocking down cells.** Relative mRNA expression levels of *AR* in 22Rv1-RR cells (A) and DU145 (B) cells upon MUC1 siRNA silencing with three different oligos (13.1 to 13.3) and the respective negative control. Results are presented as mean ± SD of at least 3 independent experiments. *GUSB* was used as reference gene for normalization.

**Supplementary fig. 5. Impact of *AR* and *MUC1* knockdown for radiotherapy responsiveness.** Cell survival fraction (SF) of 22Rv1-RR (A) and 22Rv1-P (B) upon MUC1 and AR siRNA silencing, respectively with three different oligos (13.1 to 13.3) and the respective negative control, represented through linear-quadratic model (LQ = (S=e ^– (xD + BD2)^)), ***, *p value <0.001* **** *p value <0.0001*. Original 22Rv1-P and 22Rv1-RR cell survival curves were used as a reference (yellow arrow).

**Supplementary fig. 6. *Identification of 22Rv1-RR cell traits and relevant signaling pathways by Mass spectrometry*.** A) Heatmap representing the relative abundance of specific PCa-related peptides identified in 22Rv1-P (parental) and -RR (radioresistant) samples using mass spectrometry (LC-MS) analysis. Rows represented the detected peptides and columns represent each analyzed samples, 22Rv1-P in light blue and 22Rv1-RR in light red, in independent triplicates. The color intensity of each square reflects the relative abundance of a specific proteins. The range is indicated by the scale bar with red indicating higher abundance, blue indicating lower abundance and white representing no abundance detected. B) Focal adhesion pathway and C) cell adhesion molecules found in 22Rv1-RR cells compared to 22Rv1-P cells fraction. Red color represents higher abundance, and green color represents lower abundance relative to the reference group. Data retrieved from KEGG (Kyoto Encyclopedia of Genes and Genomes). D) Volcano plot depicting log_2_ scale fold change versus negative log_10_-transformed *p-value* for all identified peptides, highlighting VIM and CDH3. Light Red dots indicate peptides with statistically significant up-regulation (p-value < 0.05, fold change > 2), and light blue dots indicate peptides with statistically significant down-regulation (p-value < 0.05, fold change < -2). Sample RR (22Rv1-RR cells in triplicates) was compared with samples P (22Rv1-P cells in triplicates). E) Total protein levels of VIM (57 KDa) and PCAD (28 kDa) in 22Rv1-P and -RR cells. β-actin (42kDa) was used as loading control. Images were acquired by Chemidoc detection system (Bio-Rad, USA). F) Total protein levels of VIM (57 KDa) in PC3-NC (negative control) and PC3-AR cells. β-actin (42kDa) was used as loading control. Images were acquired by Chemidoc detection system (Bio-Rad, USA). G) Bar chart showing significantly enriched pathways related with cell lines in 22Rv1-RR cells identified through the proteomic analysis for the differential expressed peptides between 22Rv1-P (parental) and -RR (radioresistant) cells. Higher bar heights indicate stronger enrichment. Source: Online Enricher library.

**Supplementary fig. 7. Morphometrics in 22Rv1-P and -RR cells.** A) and B) Bright-field microscopy images of 22Rv1-P and 22Rv1-RR cells, respectively. Images were taken at 10x of magnification (scale bar=100μm). C) - H) Morphometric parameters regarding 22Rv1-RR and 22Rv1-P cells, such as area in pixel^2^, perimeter in pixel, circularity skewness, roundness and solidity, respective. All the parameters were analyzed using ImageJ tools. **** *p value <0.0001*.

**Supplementary fig. 8.** A) Bar chart showing significantly downregulated pathways and B) tissue related peptides in 22Rv1-RR cells identified through the proteomic analysis for the differential expressed peptides between 22Rv1-P (parental) and -RR (radioresistant) cells. Higher bar heights indicate stronger enrichment. Source: Online Enricher library. C) Heatmap representing the relative abundance of peptides identified in 22Rv1-P (parental) and -RR (radioresistant) samples using Gene set enrichment analysis (GSEA), molecular signature database (MsigDB) for “androgen receptor network” Human gene set. Rows represented the detected peptides and columns represent each analyzed samples, 22Rv1-P in light blue and 22Rv1-RR in light red, in independent triplicates.

**Supplementary fig. 9. TYE^TM^ 563 DS Transfection Control.** Bright field, red fluorescence and merged images for the TYE^TM^ 563 DS Transfection Control in 22rv1-P, 22Rv1-RR and DU145 cell lines. Red dots represent the cells that incorporated the dye. Images were acquired at 20x magnification (scale bar= 50μm).

**Supplementary fig. 10. Uncropped and original Western blot images from the main figures.** All the figures and the respective panels including Western blot images are represented in this figure. Red square represents the cropped section to the main figure. IB, Immunoblot.
