# Supplementary figures and images for: Decoding MUC1 and AR axis in a radiation-induced neuroendocrine prostate cancer cell-subpopulation unveils novel therapeutic targets
Source: Cell Death Discov. 2025 Jul 3;11:306. doi: 10.1038/s41420-025-02597-4 (PMC12229644; doi:10.1038/s41420-025-02597-4)

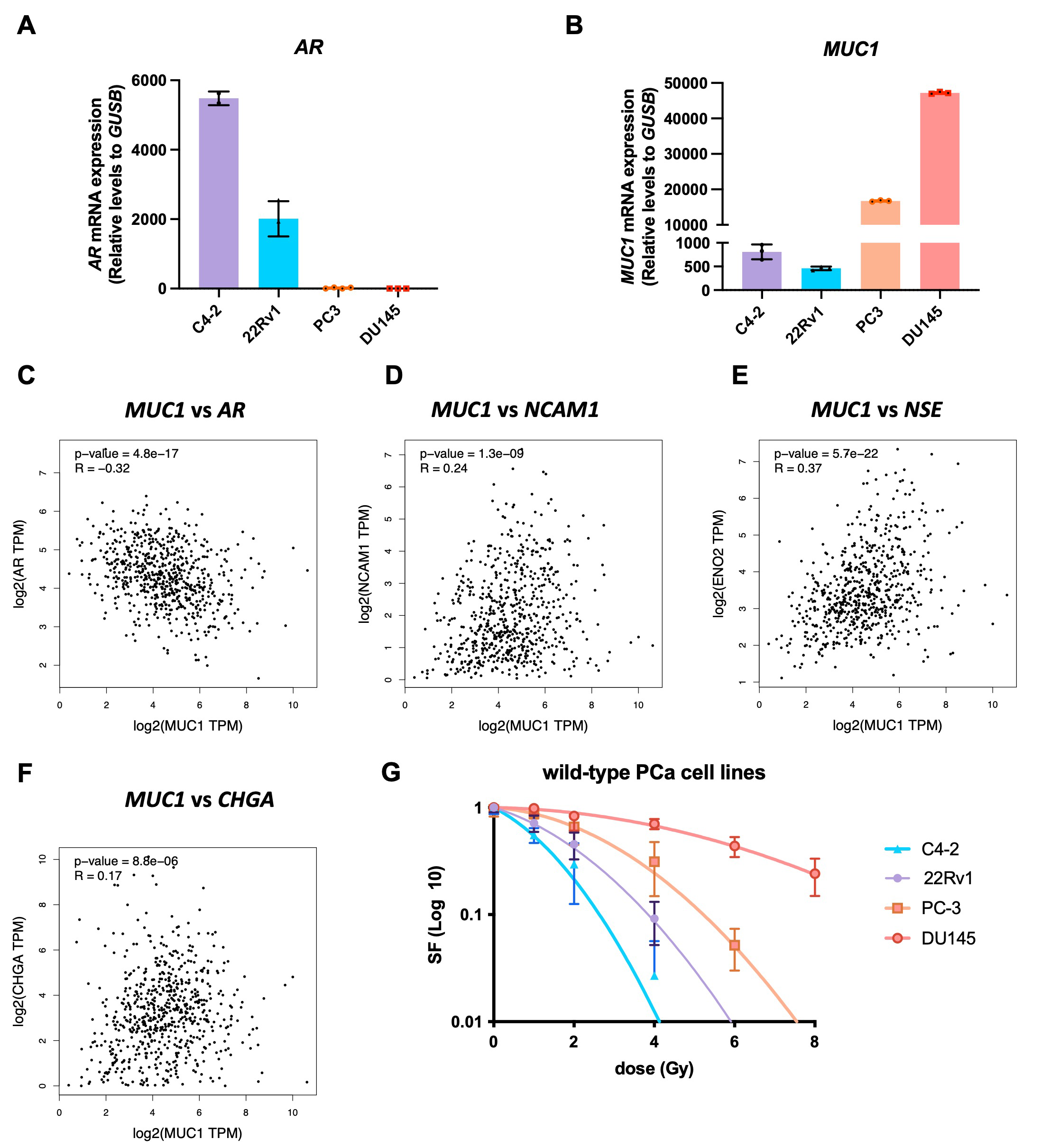

Supplement: Supplementary file 2 — Supplementary figure 1 [file 41420_2025_2597_MOESM2_ESM.tif]

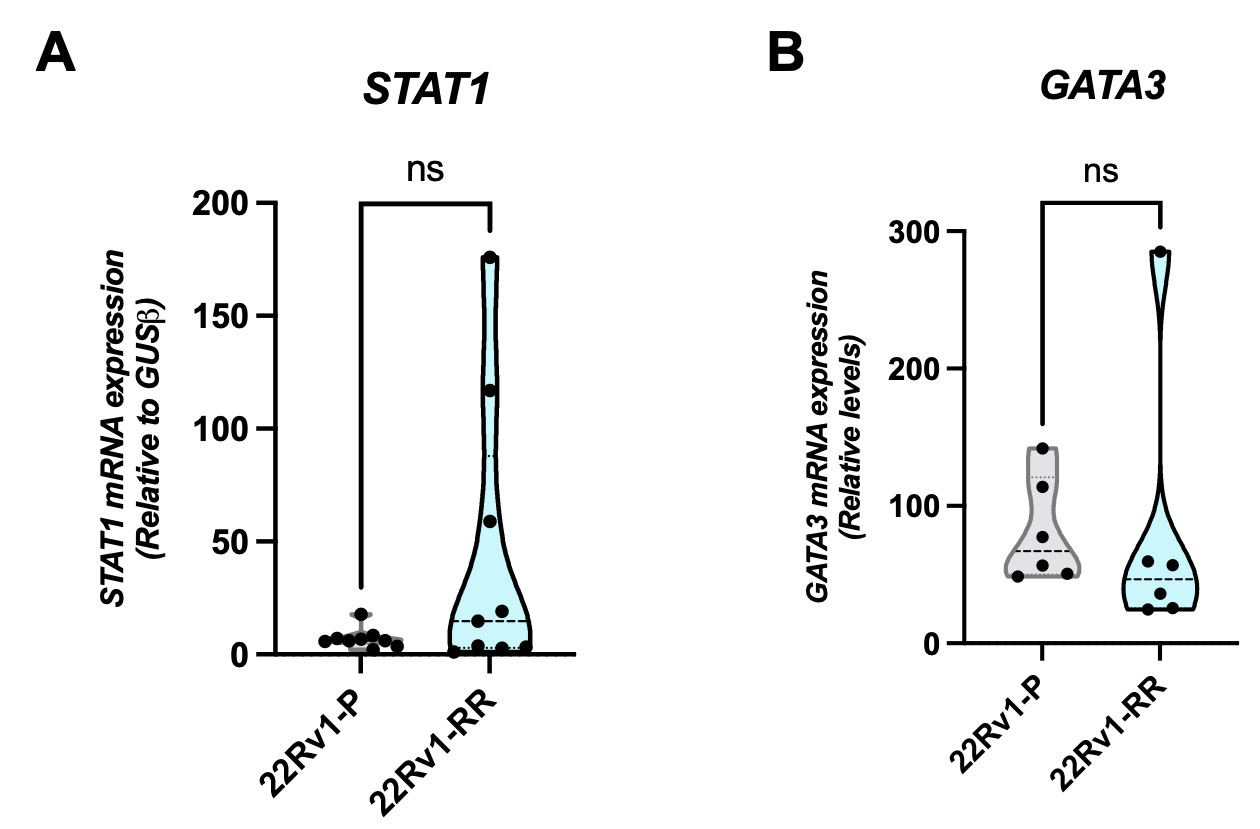

Supplement: Supplementary file 3 — Supplementary figure 2 [file 41420_2025_2597_MOESM3_ESM.tif]

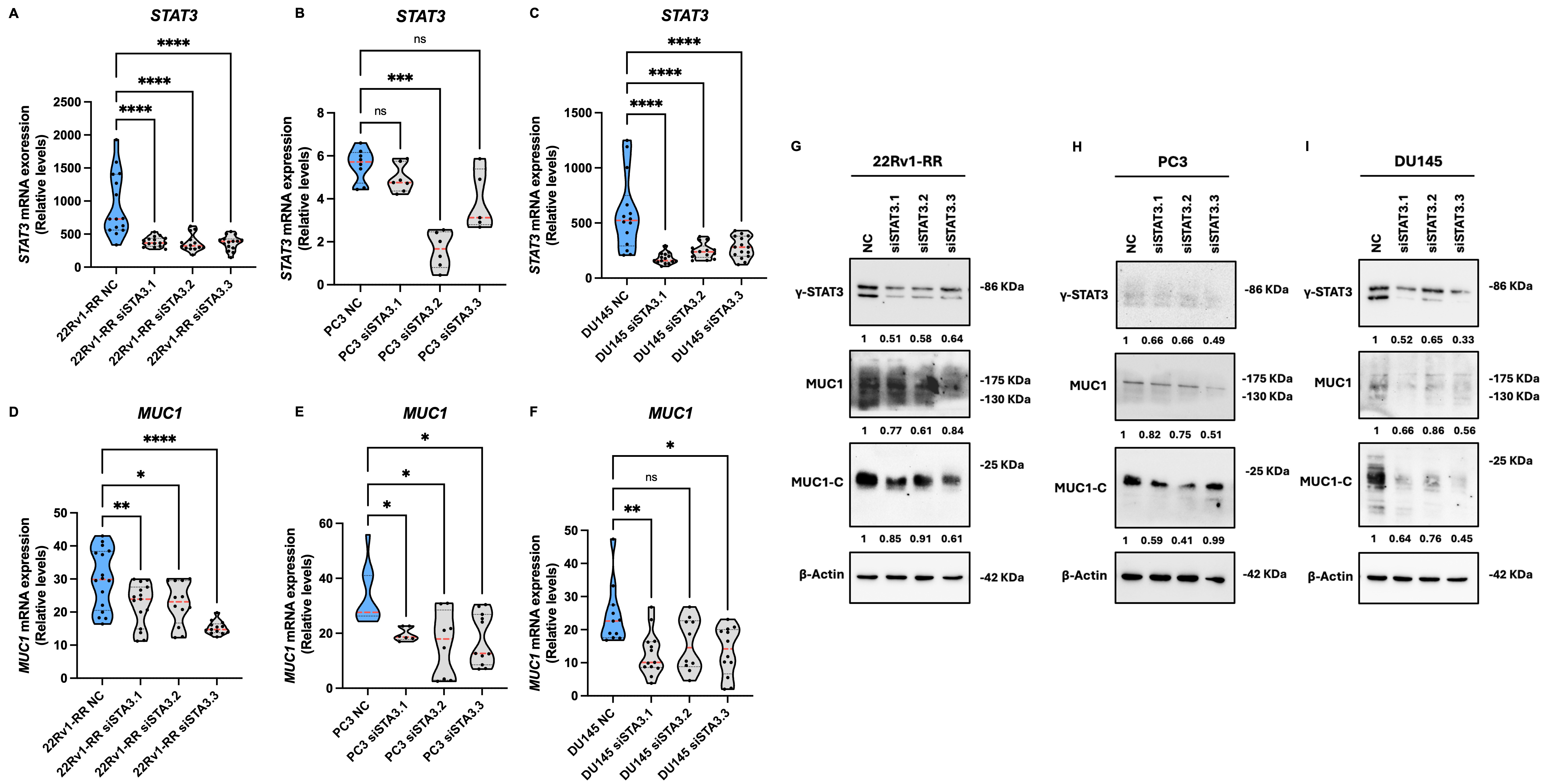

Supplement: Supplementary file 4 — Supplementary figure 3 [file 41420_2025_2597_MOESM4_ESM.tif]

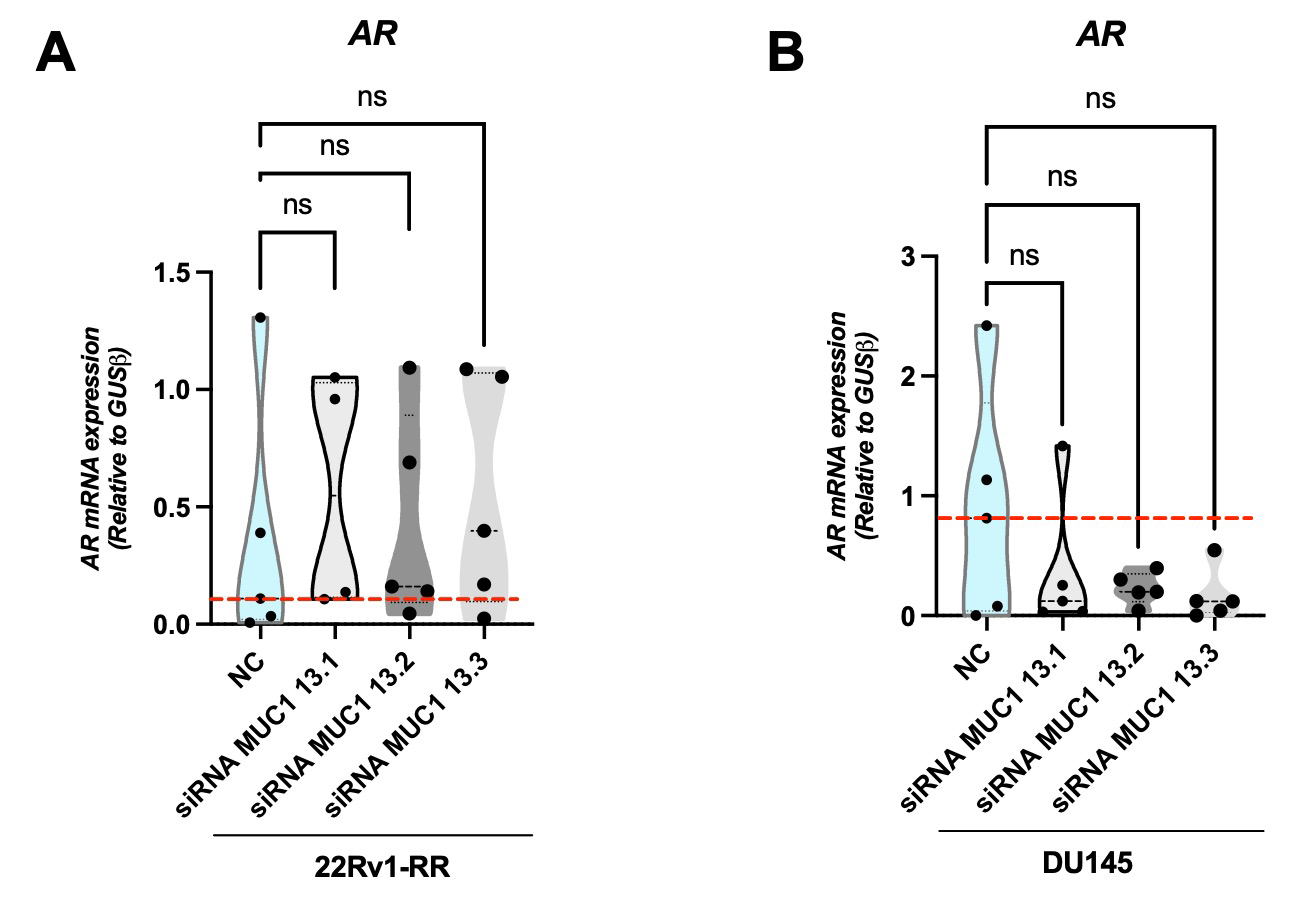

Supplement: Supplementary file 5 — supplementary figure 4 [file 41420_2025_2597_MOESM5_ESM.tif]

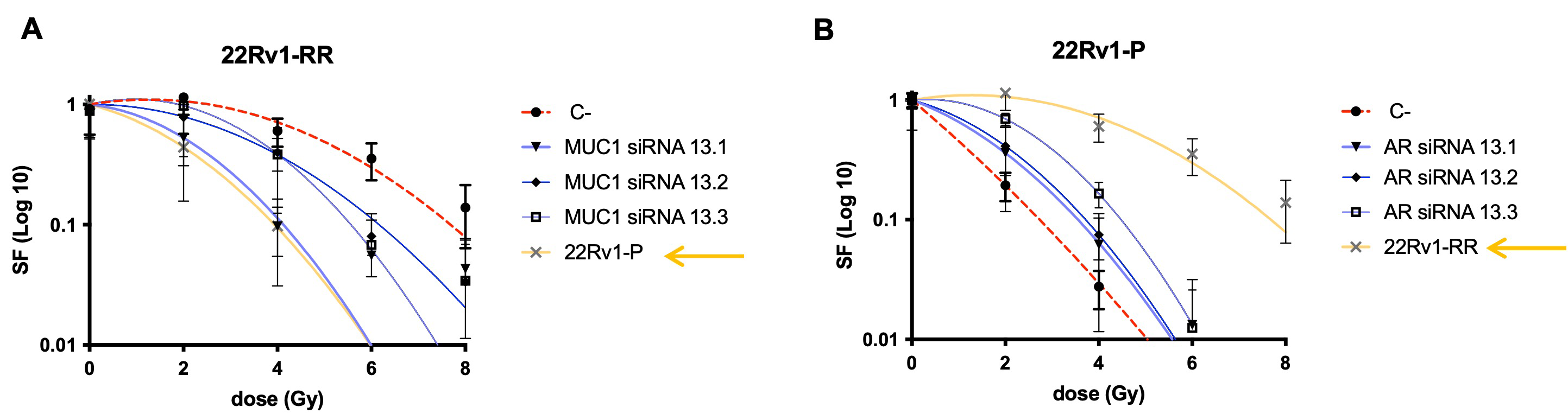

Supplement: Supplementary file 6 — supplementary figure 5 [file 41420_2025_2597_MOESM6_ESM.tif]

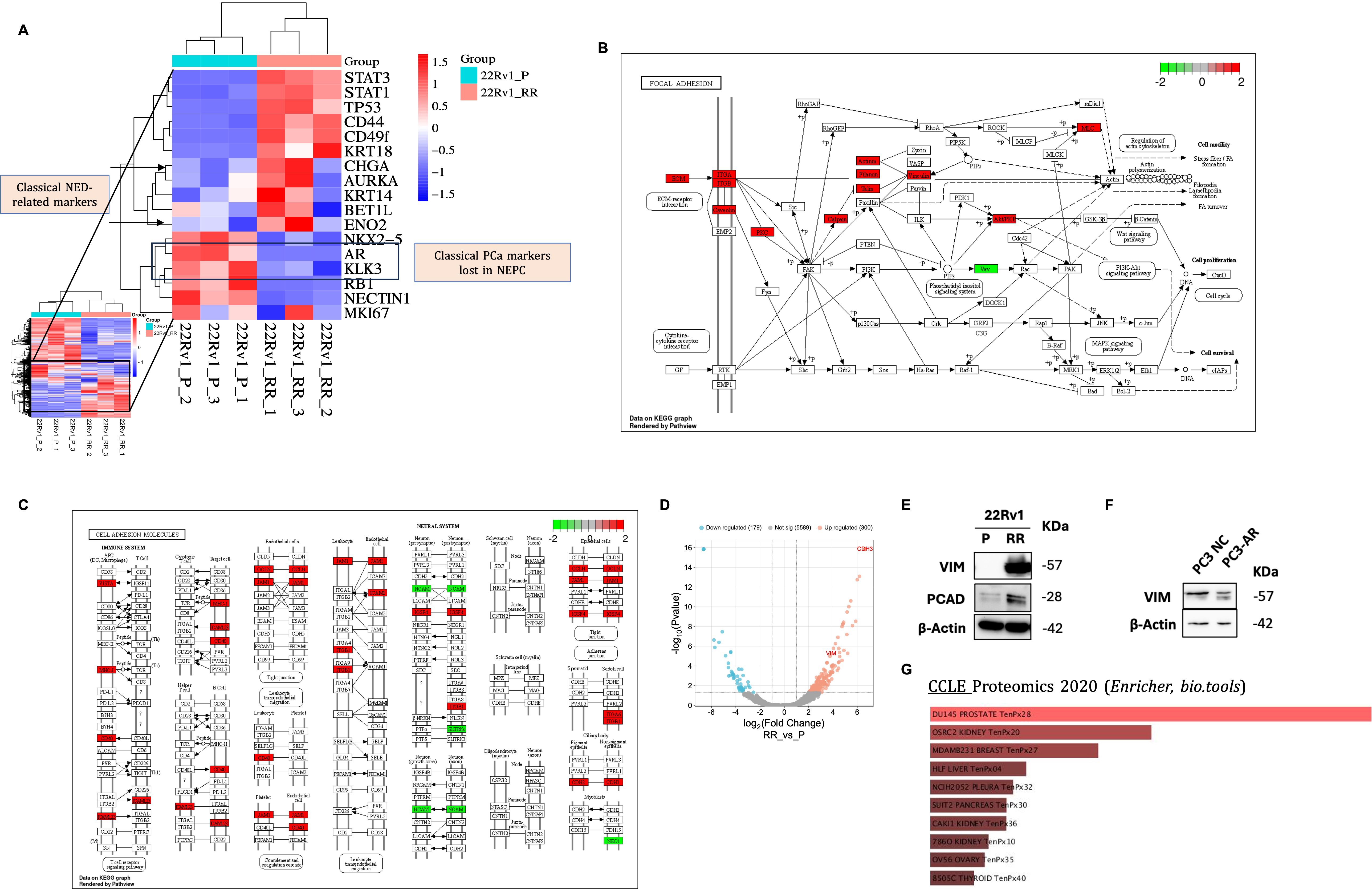

Supplement: Supplementary file 7 — supplementary figure 6 [file 41420_2025_2597_MOESM7_ESM.tif]

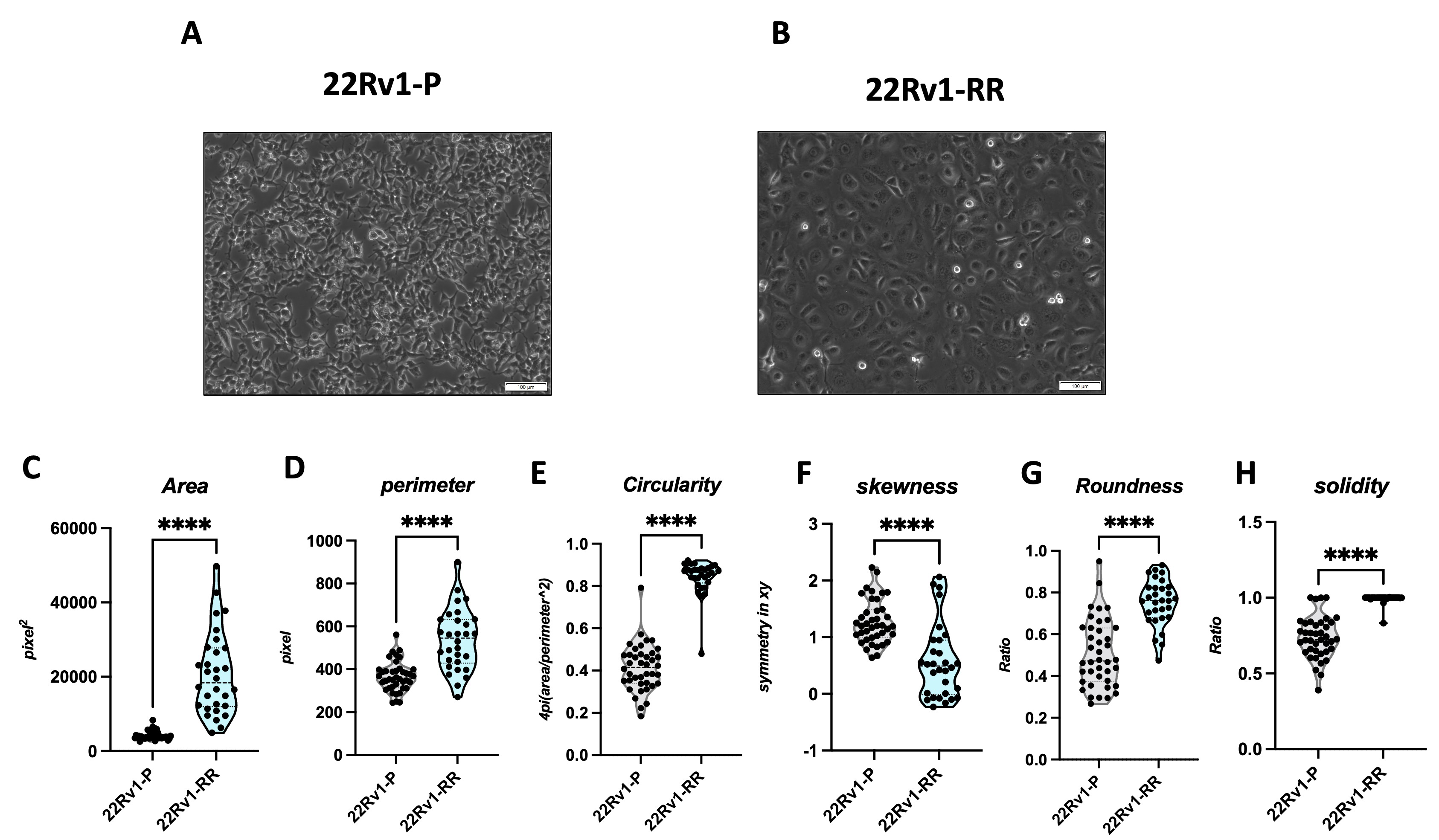

Supplement: Supplementary file 8 — supplementary figure 7 [file 41420_2025_2597_MOESM8_ESM.tif]

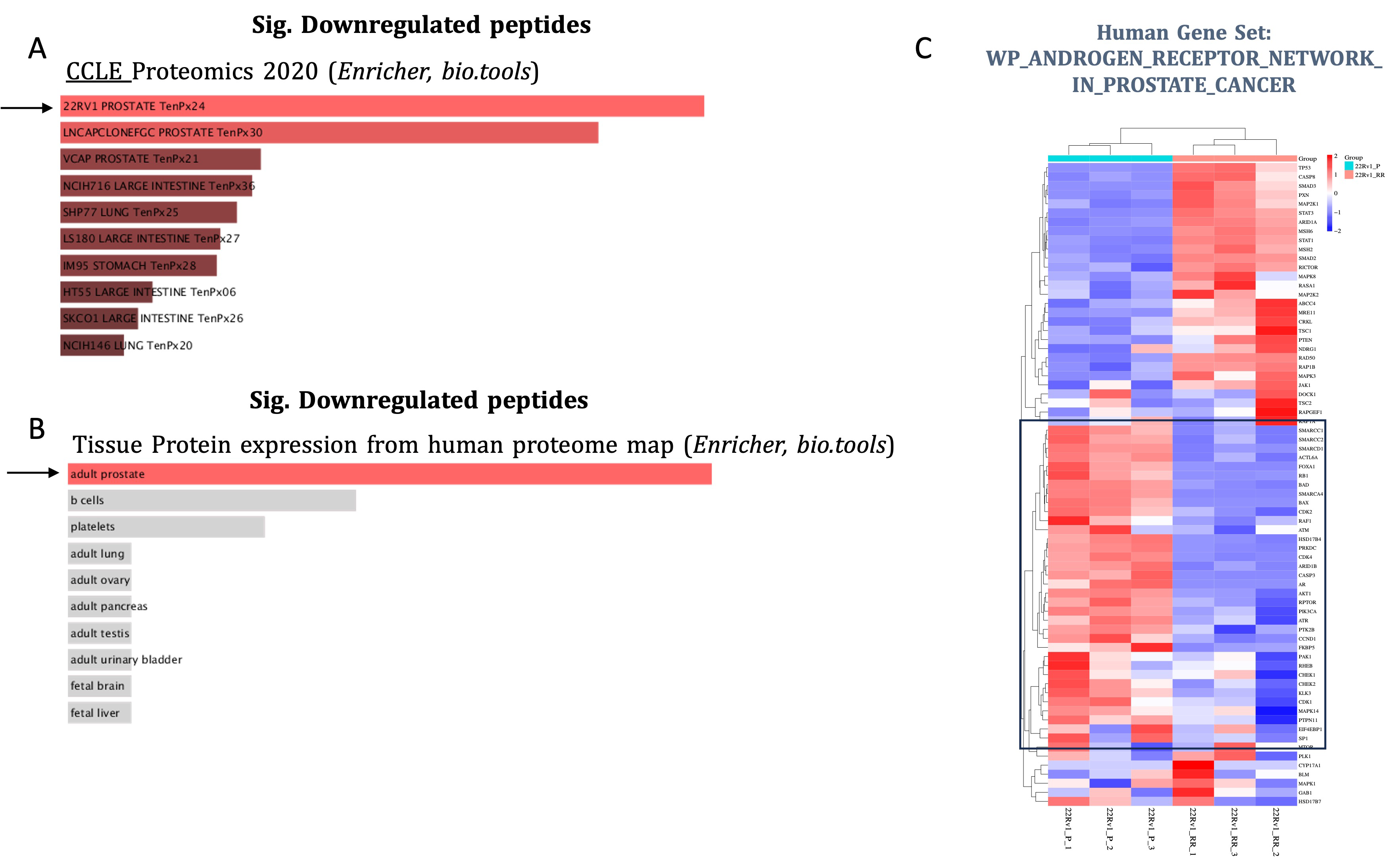

Supplement: Supplementary file 9 — supplementary figure 8 [file 41420_2025_2597_MOESM9_ESM.tif]

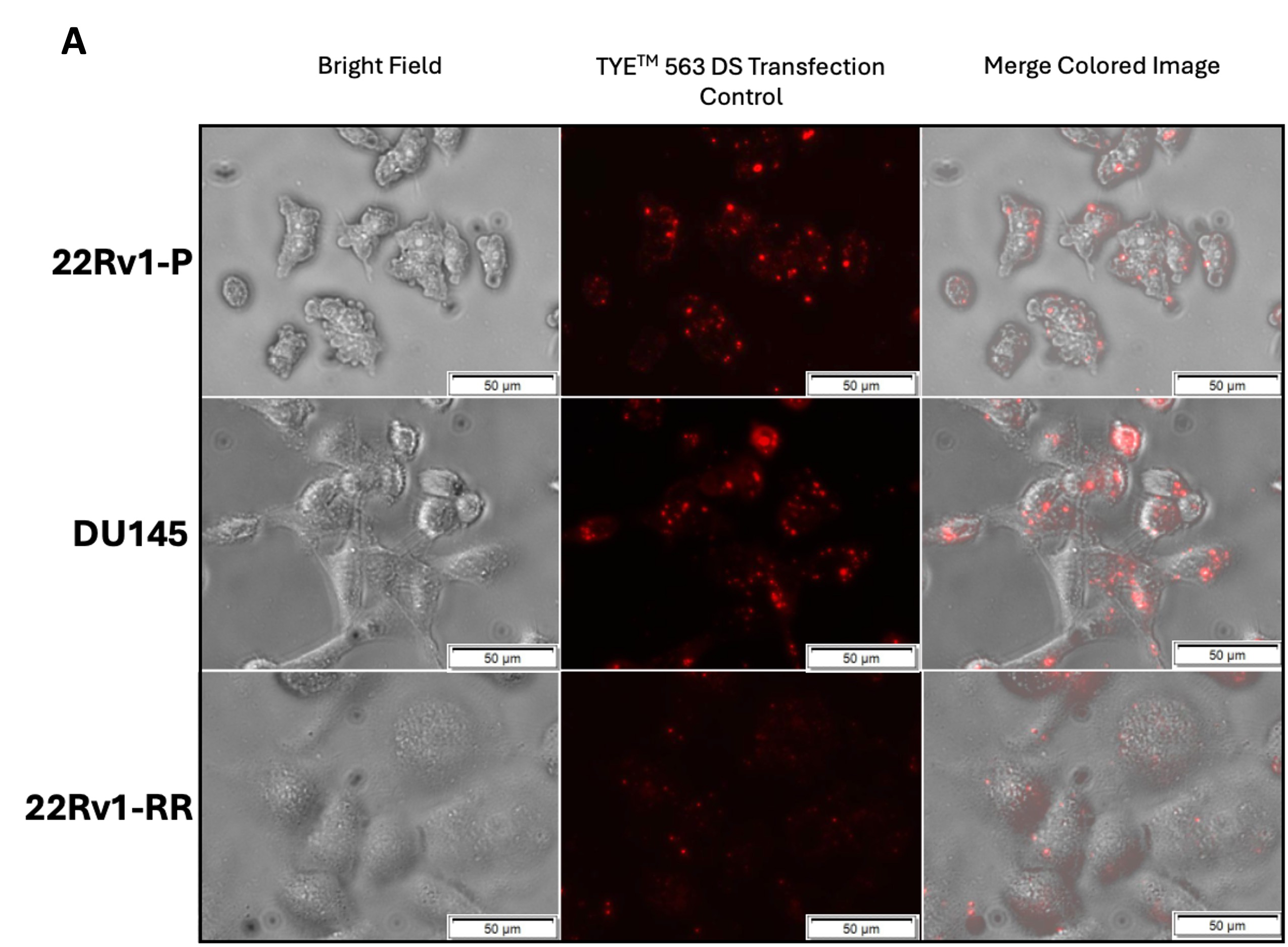

Supplement: Supplementary file 10 — supplementary figure 9 [file 41420_2025_2597_MOESM10_ESM.tif]
