# Supplementary material for: Decoding MUC1 and AR axis in a radiation-induced neuroendocrine prostate cancer cell-subpopulation unveils novel therapeutic targets
Source: Cell Death Discov. 2025 Jul 3;11:306. doi: 10.1038/s41420-025-02597-4 (PMC12229644; doi:10.1038/s41420-025-02597-4)
Supplement: Supplementary file 13 — Supplementary tables [file 41420_2025_2597_MOESM13_ESM.docx]

**Supplementary Table 1.**

| # | TF | Target | Mode of regulation | References  (PMID) |
| --- | --- | --- | --- | --- |
| 1 | AR | MUC1 | Repression | 21308711 |
| 2 | GATA1 | MUC1 | Unknown | 17078870 |
| 3 | GATA3 | MUC1 | Activation | 17078870 |
| 4 | HIF1A | MUC1 | Unknown | 19141292 |
| 5 | PIAS1 | MUC1 | Repression | 17717071 |
| 6 | PIAS2 | MUC1 | Repression | 17717071 |
| 7 | PIAS3 | MUC1 | Repression | 17717071 |
| 8 | PIAS4 | MUC1 | Repression | 17717071 |
| 9 | STAT1 | MUC1 | Activation | 17151127 |
| 10 | STAT3 | MUC1 | Activation | 17151127; 19578748; 21325207 |
| 11 | ZEB1 | MUC1 | Repression | 12161443 |

Adapted from TRRUST v2: an expanded reference database of human and mouse transcriptional regulatory interactions.Nucleic Acids Research 26 Oct, 2017

**Supplementary Table 2.**

| **TF** | **Overlapped target genes with AR** | ***P value*** |
| --- | --- | --- |
| STAT3 | 13 | 3.6634644702575e-11 |
| GATA3 | 7 | 2.38438664511143e-08 |
| STAT1 | 5 | 0.000253113949450557 |

Adapted from TRRUST v2: an expanded reference database of human and mouse transcriptional regulatory interactions. Nucleic Acids Research 26 Oct, 2017

**Supplementary Table 3. Primer sequences**

| Gene | Foward | Reverse | Annealing temperature |
| --- | --- | --- | --- |
| AR-FL | 5’ AACAGAAGTACCTGTGCGCC 3’ | 5’TTCAGATTACCAAGTTTCTTCAGC 3’ | 60ºC |
| MUC1 | 5 ́TACCGATCGTAGCCCCTATG 3’ | 5’ CTCACCAGCCCAAACAGG 3’ | 60ºC |
| STAT3 | 5’ CAGTCAGTGACCAGGCAGAA 3’ | 5’ AGTCAGCCAGCTCCTCGTC 3’ | 60ºC |
| STAT1 | 5’ TCTCATGCTCCTGCAAGCTA 3’ | 5’ CCTCCCATCAACTGGACAAC 3’ | 62ºC |
| GATA3 | 5’CAGACCACCACAACCACACTCT 3’ | 5’ GGATGCCTCCTTCTTCATAGTCA 3’ | 60ºC |

FL, Full length.
